# Integrative single-cell, spatial, and bulk transcriptomics reveal an FMR1–FTO axis linked to the immune-excluded phenotype in gastric cancer

**DOI:** 10.3389/fimmu.2026.1713267

**Published:** 2026-03-09

**Authors:** Chenxi Mao, Miao Zhang, Kangjie Zhou, Yidong Hong, Yiqian Han, Luming Zhao, Mingtong Liang, Jingzhou Zhang, Nan Hu, Fenglei Wu

**Affiliations:** 1 The Affiliated Lianyungang Hospital of Xuzhou Medical University, Lianyungang, China; 2 Lianyungang Clinical College of Nanjing Medical University, Lianyungang, China

**Keywords:** FMR1, FTO, gastric cancer, immune exclusion, immune phenotype, single-cell transcriptomics, spatial transcriptomics

## Abstract

**Background:**

Immune exclusion is a major barrier to immune checkpoint inhibitor (ICI) efficacy in gastric cancer, yet the spatial mechanisms by which m^6^A regulators drive this phenotype remain unclear.

**Methods:**

We integrated single-cell RNA-seq, spatial transcriptomics, and functional assays to map the gastric cancer microenvironment and derived an m^6^A regulator score to quantify the spatial coupling between m^6^A patterns and immune exclusion.

**Results:**

CAFs emerged as a central hub that excluded T cells via collagen–integrin interactions and MIF signaling, forming a CAF-defined collagen barrier that impeded CD8^+^ T-cell entry into the tumor core. In immune-excluded samples, m^6^A regulator scores were selectively elevated in tumor nests compared with surrounding stroma. Across five bulk transcriptomic datasets, this phenotype was associated with reduced predicted ICI responsiveness, activation of chemotherapy resistance programs, and poor survival. Mechanistically, FTO was highly expressed in the immune-excluded phenotype and correlated with stromal activation and T-cell exclusion. Serial immunohistochemistry and multiplex immunofluorescence revealed high FMR1 protein expression in immune-excluded tumors, with CD8^+^ T cells largely confined to the stroma. Cycloheximide (CHX) chase and MG132 treatment showed that FMR1 depletion reduced FTO protein abundance and accelerated FTO turnover in an MG132-sensitive manner, consistent with a post-translational regulatory relationship.

**Conclusions:**

Collectively, our data support an FMR1–FTO module associated with the immune-excluded phenotype and nominate this axis as a potential vulnerability for disrupting stromal immune barriers. The FMR1–FTO axis may represent a candidate target for strategies aimed at relieving immune exclusion and improving immunotherapy sensitivity.

## 1 Introduction

Gastric cancer (GC) ranks fifth in both incidence and mortality among malignancies worldwide, characterized by profound molecular and phenotypic heterogeneity (1, 2). Historically, while the Lauren classification and molecular subtypes defined by The Cancer Genome Atlas (TCGA)—such as EBV, MSI, GS, and CIN—have greatly enriched our understanding of GC biology, their translational utility in guiding precision oncology, particularly in predicting responses to immunotherapy, remains limited (3, 4).

Although immune checkpoint blockade (ICB) has reshaped the treatment landscape for advanced GC (5–8), clinical reality remains grim: the objective response rate (ORR) hovers between 10% and 26%, with the vast majority of patients encountering primary resistance or treatment failure (7–10). To address this heterogeneity, the concept of tumor immune phenotypes has emerged, among which the “immune-excluded” phenotype is particularly prevalent in GC (11). In this microenvironment, despite the presence of abundant immune infiltration, effector T cells are physically sequestered from tumor nests by a dense stromal barrier—composed primarily of cancer-associated fibroblasts and extracellular matrix deposition—creating a distinct “cold tumor” state. This unique spatial architecture severely restricts anti-tumor immunity and represents a critical bottleneck in overcoming resistance to immunotherapy.

While the abundance of immune infiltration is closely linked to patient prognosis (12–14), the cellular architecture and molecular drivers of the GC tumor immune microenvironment (TIME) remain incompletely mapped. Specifically, a robust immune classification system based on spatial features to guide stratified treatment is still lacking (15, 16). The advent of high-dimensional technologies, such as single-cell RNA sequencing (scRNA-seq), spatial transcriptomics, and multiplex immunofluorescence (mIF), has made it possible to dissect TIME heterogeneity and intercellular communication at single-cell resolution and spatial dimensions, providing unprecedented opportunities to uncover the drivers of immune exclusion (17–21).

N6-methyladenosine (m6A), the most prevalent internal modification in eukaryotic mRNA (22, 23), plays a pivotal role in tumor progression (24). Although the effects of m^6^A regulators on tumor cell-intrinsic malignancy have been widely reported (25), a systemic understanding of how they induce the immune-excluded phenotype by remodeling the TIME is lacking. The m^6^A demethylase FTO has been confirmed to promote immune evasion in various cancers (26, 27) and is highly expressed in the immune-excluded phenotype of GC (28); however, the upstream mechanism maintaining high FTO protein stability in this context remains unclear. Notably, a recent study published in Science identified the RNA-binding protein FMRP (encoded by FMR1) as a molecular switch mediating the transition between “cold” and “hot” tumors (29). Inspired by this, we hypothesized an intrinsic link between FMR1 and the immune-excluded phenotype in GC: Could FMR1 reshape the immune landscape through a non-canonical mechanism—specifically, by directly regulating FTO stability at the protein level?

Based on this hypothesis, this study aims to systematically map the panoramic landscape of the GC immune microenvironment by integrating multi-omics analysis. We first established a robust immune classification system based on spatial features, focusing on molecular characteristics significantly associated with the “immune-excluded” phenotype. Furthermore, we dissected the core regulatory network of the FMR1-FTO axis from the perspective of post-translational modification. By revealing a proteasome-dependent mechanism whereby FMR1 stabilizes FTO protein, our findings not only provide a rational molecular explanation for the aberrant accumulation of FTO in specific immune phenotypes but also highlight a novel therapeutic target with potential clinical value for overcoming immunotherapy resistance in gastric cancer.

## 2 Materials and methods

### 2.1 Data acquisition and preprocessing

Single-cell RNA sequencing (scRNA-seq) data comprising 26 gastric cancer (GC) and 10 normal tissue samples were retrieved from the GEO database (GSE183904). Quality control was rigorously performed using the Seurat package (v5.0.1) in R (v4.4.1). Low-quality cells (detected genes <200 or >5,000; mitochondrial genes >10%) and low-abundance genes were excluded, yielding 117,965 high-quality cells for downstream analysis. Spatial transcriptomics (ST) data (GSE251950, 10x Visium platform) were processed using Seurat and STUtility, with normalization via SCTransform. To resolve spatial cellular composition, scRNA-seq-annotated cell types (tumor cells, fibroblasts, and T cells) were mapped onto ST spots using anchor-based integration (FindTransferAnchors and TransferData functions), generating a prediction score matrix for cell type abundance. Additionally, five independent GC transcriptomic cohorts (GSE15459, GSE34942, GSE57303, GSE62254, GSE84437) with clinical follow-up data were curated. Affymetrix data were preprocessed using the Robust Multi-array Average (RMA) algorithm. Batch effects across cohorts were corrected using the ComBat algorithm (sva package) and normalizeBetweenArrays function (limma package) to ensure comparability (**Supplementary Figures S1**, **S2**).

### 2.2 Cell enrichment analysis

To investigate biological functions across different cell types, we identified differentially expressed genes for each cluster and retained those exhibiting significant positive fold-change. Using the clusterProfiler framework, we performed Gene Ontology (GO) biological process enrichment analysis on the top 20 marker genes within each cluster, implementing multiple testing correction (corrected P-value < 0.05). Enrichment results were visualized via composite graphs and heatmaps, synthesising key pathways across cell types.

### 2.3 Cell-cell communication and spatial analysis

Intercellular communication networks were inferred using CellChat (v1.6.1) based on known ligand-receptor interactions, with a specific focus on signaling axes between cancer-associated fibroblasts (CAFs) and T cells. Spatially, composite RGB images were constructed to visualize the topological relationship among tumor nests (mapped to red), stromal barriers (green), and T cells (blue). To assess the epigenetic state within the TME, a composite m^6^A regulator scores was calculated for each spatial spot using the AddModuleScore function, based on a set of 21 m^6^A regulators (30) (writers, erasers, and readers). Spatial spots were segmented into “tumor nests” or “stroma” based on cell type prediction scores (threshold > 0.1), and m^6^A regulator scores distributions were compared using the Wilcoxon rank-sum test.

### 2.4 m^6^A subtyping and TME evaluation

Unsupervised consensus clustering was performed based on the expression profiles of 21 m^6^A regulators in the merged GEO cohort using the ConsensusClusterPlus package (1,000 iterations) to identify robust molecular subtypes. The relative abundance of immune and stromal cell populations was quantified via Single-Sample Gene Set Enrichment Analysis (ssGSEA), (**Supplementary Table 1**), and TME purity was assessed using the ESTIMATE algorithm. Weighted Gene Co-expression Network Analysis (WGCNA) was employed to identify gene modules highly correlated with specific clusters (Cluster 2; correlation > 0.61, P < 0.001), followed by KEGG pathway enrichment. Immune evasion characteristics and potential responses to immunotherapy were evaluated using the Tumor Immune Dysfunction and Exclusion (TIDE) algorithm.

### 2.5 Clinical correlation and drug sensitivity

The independent prognostic value of m^6^A phenotypes was assessed via univariate and multivariate Cox regression analyses. A nomogram integrating molecular subtypes with clinicopathological variables was constructed, and its predictive performance was validated using time-dependent ROC and calibration curves. Chemotherapeutic response (IC50) for eight common drugs (e.g., 5-Fluorouracil, Paclitaxel, Cisplatin) was predicted using the pRRophetic package (31) based on the GDSC database.

### 2.6 Clinical specimens and immunohistochemistry

A cohort of 70 formalin-fixed paraffin-embedded (FFPE) gastric cancer tissues from patients who underwent radical gastrectomy in 2020 (without preoperative chemoradiotherapy) was retrospectively enrolled. Serial sections (4 μm) were deparaffinized, rehydrated, and subjected to heat-induced antigen retrieval in citrate buffer (32). After blocking endogenous peroxidase and non-specific binding, sections were incubated overnight at 4 °C with primary antibodies against FMR1 (1:200) and CD8 (1:200). Following secondary antibody incubation, signals were visualized using DAB and counterstained with hematoxylin. Staining was evaluated by two pathologists in a blinded manner using a semi-quantitative scoring system (staining intensity × percentage of positive cells); a total score ≥5 was defined as moderate-to-strong positivity.

### 2.7 Multiplex immunofluorescence staining

Multiplex immunofluorescence was performed on FFPE gastric cancer tissues (n = 10) using a TSA-based sequential staining protocol. After deparaffinization, antigen retrieval and blocking, slides were incubated with primary antibodies against FMR1 (rabbit; Abcam, ab17722, 5µg/mL), Collagen IV (rabbit; Abcam, ab6586, 1:400) and CD8a (mouse; Proteintech, 66868-1-Ig, 1:400). For each target, an HRP-conjugated species-specific secondary antibody was applied followed by fluorophore-tyramide incubation. Between cycles, heat-mediated stripping (microwave/antigen retrieval) was performed to remove the bound primary/secondary antibodies while preserving the covalently deposited tyramide signal, enabling detection of multiple targets including two rabbit-derived antibodies without cross-reactivity. Nuclei were counterstained with DAPI (33).

Collagen IV was interpreted as a functional readout of ECM-rich stromal barrier structures rather than a CAF-specific marker.

### 2.8 Cell culture and stable transfection

Human GC cell lines (NCI-N87, AGS, HGC-27) and the normal gastric epithelial cell line (GES-1) were obtained from the Cell Bank of the Chinese Academy of Sciences (Shanghai, China). HEK-293T cells were used for viral packaging. All cells were cultured in RPMI-1640 or DMEM (for HEK-293T) supplemented with 10% fetal bovine serum (FBS) and 1% penicillin-streptomycin at 37 °C in a humidified 5% CO_2_ atmosphere (34). Short hairpin RNAs (shRNAs) targeting FMR1 were synthesized by Tsingke Biological Technology (China) (sequences listed in **Table 1**). For lentiviral production, a three-plasmid packaging system comprising the target plasmid (pLVX-shRNA-Puro-FMR1), psPAX2, and pMD2.G (ratio 4:3:1) was co-transfected into HEK-293T cells (70–80% confluence) using Lipofectamine™ 2000 (Invitrogen). Viral supernatants were collected 48 h post-transfection and filtered through a 0.45 μm membrane. NCI-N87 cells were infected with viral particles in the presence of 8 μg/mL Polybrene. Knockdown efficiency was verified by Western blotting 48 h post-infection.

**Table 1:** Sequences of shRNAs targeting FMR1.

Name	Type	Target(5′→3′)	shRNA hairpin sequence (5′→3′)
NC	Negative control	ACTACCGTTGTTATAGGTGT	gatcgACTACCGTTGTTATAGGTGTCTCGAGACACCTATAACAACGGTAGTTTTTTT
sh*FMR1*-1	shRNA	GCGTTTGGAGAGATTACAAAT	gatcgGCGTTTGGAGAGATTACAAATCTCGAGATTTGTAATCTCTCCAAACGCTTTTTT
sh*FMR1*-2	shRNA	GCCAGAAGACTTACGGCAAAT	gatcgGCCAGAAGACTTACGGCAAATCTCGAGATTTGCCGTAAGTCTTCTGGCTTTTTT
sh*FMR1*-3	shRNA	CGAGATTTCATGAACAGTTTA	gatcgCGAGATTTCATGAACAGTTTACTCGAGTAAACTGTTCATGAAATCTCGTTTTTT

### 2.9 Western blotting

Cells were lysed in RIPA buffer containing protease inhibitors on ice. Protein concentration was quantified using a BCA Protein Assay Kit (NCM Biotech). Equal amounts of protein were resolved by SDS-PAGE and transferred onto PVDF membranes (Millipore). After blocking for 10 min at room temperature, membranes were incubated overnight at 4 °C with primary antibodies: anti-FMR1 (ab17722,1:1000, Abcam), anti-FTO (ab124892,1:1000,Abcam), anti-Flag (1:20,000, Proteintech), and anti-GAPDH (1:5,000, Proteintech). Membranes were then incubated with HRP-conjugated secondary antibodies for 1 h at room temperature. Protein bands were visualized using an ECL kit (35, 36) (Proteintech) and detected via a bioimaging system (Proteinsimple). Band intensity was quantified using ImageJ software, normalized to GAPDH.

### 2.10 Co-immunoprecipitation

To investigate the physical interaction between FMR1 and FTO, NCI-N87 cells expressing Flag-tagged FMR1 or GFP control were lysed in IP lysis buffer (Thermo Fisher Scientific) on ice for 30 min. Lysates were incubated overnight at 4 °C with anti-Flag magnetic beads (Selleckchem). The beads were washed three times with wash buffer, and immunocomplexes were eluted by boiling in loading buffer at 95 °C for 5 min (37). Eluates and input samples were subjected to Western blot analysis.

### 2.11 Protein stability assays

Cycloheximide (CHX) Chase Assay: NCI-N87 cells transfected with shNC or shFMR1 were treated with the protein synthesis inhibitor CHX (100 μg/mL, MCE). Cells were harvested at indicated time points (0, 3, 6, and 9 h). FTO protein levels were assessed by Western blotting to evaluate protein half-life.

Proteasome Inhibition Assay: Cells were treated with the proteasome inhibitor MG132 (10 μM, MCE) for 6 h prior to harvesting. Total protein was extracted, and FTO expression was analyzed by Western blotting to determine the involvement of the proteasomal degradation pathway.

### 2.12 Molecular docking and molecular dynamics simulations

Protein–protein docking between FMR1 (PDB: 2QND) and FTO (PDB: 3LFM) was performed using HDOCKlite v1.1, and the top-ranked model (highest confidence and lowest docking score) was further evaluated by MM/GBSA binding free-energy estimation (HawkDock) and interface interaction profiling (PLIP/PyMOL) (38). The selected complex was subjected to all-atom MD simulations in GROMACS 2022 with the AMBER14SB force field to assess structural stability (39).

### 2.13 Statistical analysis

Statistical analyses of clinical and experimental data were performed using SPSS 26.0 (IBM Corp., Armonk, NY, USA) and GraphPad Prism 9.0 (GraphPad Software, San Diego, CA, USA). Omics-related analyses (e.g., ssGSEA, ESTIMATE, WGCNA, TIDE, survival curves and nomograms) were conducted in R software (version 4.4.1; R Foundation for Statistical Computing, Vienna, Austria). Continuous variables are presented as mean ± standard deviation (SD) or median (interquartile range), as appropriate. Differences between two groups were assessed using the Student’s t test or Wilcoxon rank-sum test. Comparisons among three or more groups were performed using one-way analysis of variance (ANOVA) or the Kruskal–Wallis test. Categorical variables were compared using the χ² test or Fisher’s exact test. Overall survival was analysed using the Kaplan–Meier method and log-rank test, and hazard ratios (HRs) with 95% confidence intervals (CIs) were estimated by Cox proportional hazards regression. All P values were two-sided, and Significance markers are: ns, P ≥ 0.05; * P < 0.05; ** P < 0.01; *** P < 0.001; **** P < 0.0001.

## 3 Results

### 3.1 Construction of the single-cell transcriptomic atlas and decoding the fibroblast-T cell crosstalk network in gastric cancer

**Figure 1:**
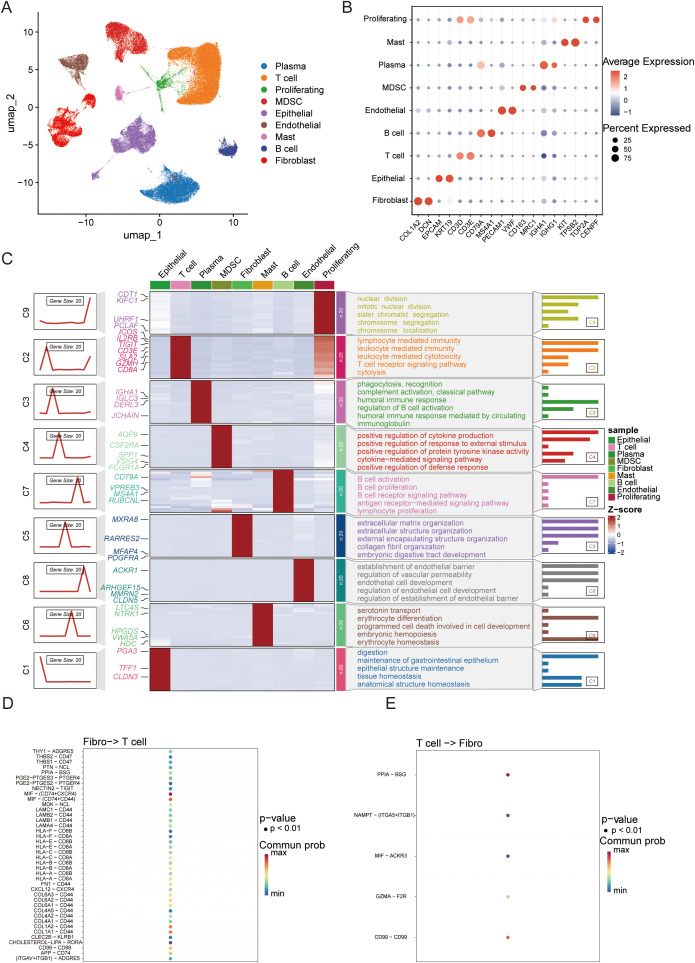
Construction of the single-cell transcriptomic atlas and decoding the fibroblast-T cell crosstalk network in gastric cancer. **(A)** UMAP visualization of 117,965 cells from gastric cancer and matched normal tissues, coloured by major cell lineages (epithelial cells, fibroblasts, endothelial cells, T cells, B cells, plasma cells, mast cells and MDSCs); **(B)** Dot plot showing the expression of canonical marker genes across major cell lineages, with dot size indicating the percentage of expressing cells and colour reflecting average expression; **(C)** Heatmap of representative marker genes and Gene Ontology biological process enrichment for each lineage, highlighting lineage-specific functional programs; **(D)** Bubble plot of significant ligand–receptor pairs mediating fibroblast-to–T cell signaling, with dot size proportional to –log10(P value) and colour indicating communication probability; **(E)** Bubble plot of major T cell–to-fibroblast ligand–receptor interactions, with dot size and colour representing statistical significance and communication probability, respectively.

To systematically deconstruct the cellular heterogeneity within the gastric cancer (GC) tumor microenvironment (TME), we performed dimensionality reduction and clustering on quality-controlled single-cell data. Based on distinct transcriptional signatures, cells were stratified into nine major lineages (**Figure 1A**), including epithelial cells, fibroblasts, endothelial cells, and diverse immune subsets (e.g., T cells, B cells, plasma cells, mast cells, and MDSCs). The accuracy of cell annotation was validated by the specific expression of canonical marker genes, such as COL1A2 for fibroblasts, EPCAM for epithelial cells, and CD3D for T cells (**Figure 1B**). Functional enrichment of differentially expressed genes (DEGs) further confirmed the biological identity of these subsets; notably, T cells were enriched in “T cell receptor signaling pathways,” while fibroblasts showed robust activity in “extracellular matrix (ECM) organization” (**Figure 1C**).

We reconstructed the intercellular communication network within the tumor microenvironment using CellChat. The analysis identified fibroblasts as the primary communication hub, establishing robust connections with T cells (**Supplementary Figures 3A, B**). Furthermore, signaling pattern analysis revealed that fibroblasts function predominantly as signal senders (e.g., via COLLAGEN and LAMININ pathways), while T cells act as the major recipients (**Supplementary Figures 3C, D**). Quantitative cellular composition analysis confirmed a dramatic tissue remodeling, characterized by a dual enrichment pattern with significant T cell expansion alongside persistently high fibroblast abundance (**Supplementary Figure 3E**).

Ligand-receptor pair analysis provided molecular insights into this crosstalk. We found that fibroblasts modulate T cells via the secretion of CXCL12, which binds to CXCR4, establishing a classic immunosuppressive axis. Furthermore, the extensive interaction between fibroblast-derived collagens and T cell integrins suggests the formation of a physical barrier (**Figure 1D**). Reciprocally, T cells feedback to fibroblasts via axes such as MIF-ACKR3 (**Figure 1E**). Collectively, these data delineate a fibroblast-dominated communication network that not only induces immunosuppression via chemokine axes (e.g., CXCL12-CXCR4) but also orchestrates physical exclusion via collagen deposition, providing crucial single-cell evidence for the formation of the “immune-excluded” phenotype.

### 3.2 Spatial transcriptomics unveils an m^6^A-associated immune-excluded phenotype characterized by fibroblast-mediated physical barriers

**Figure 2:**
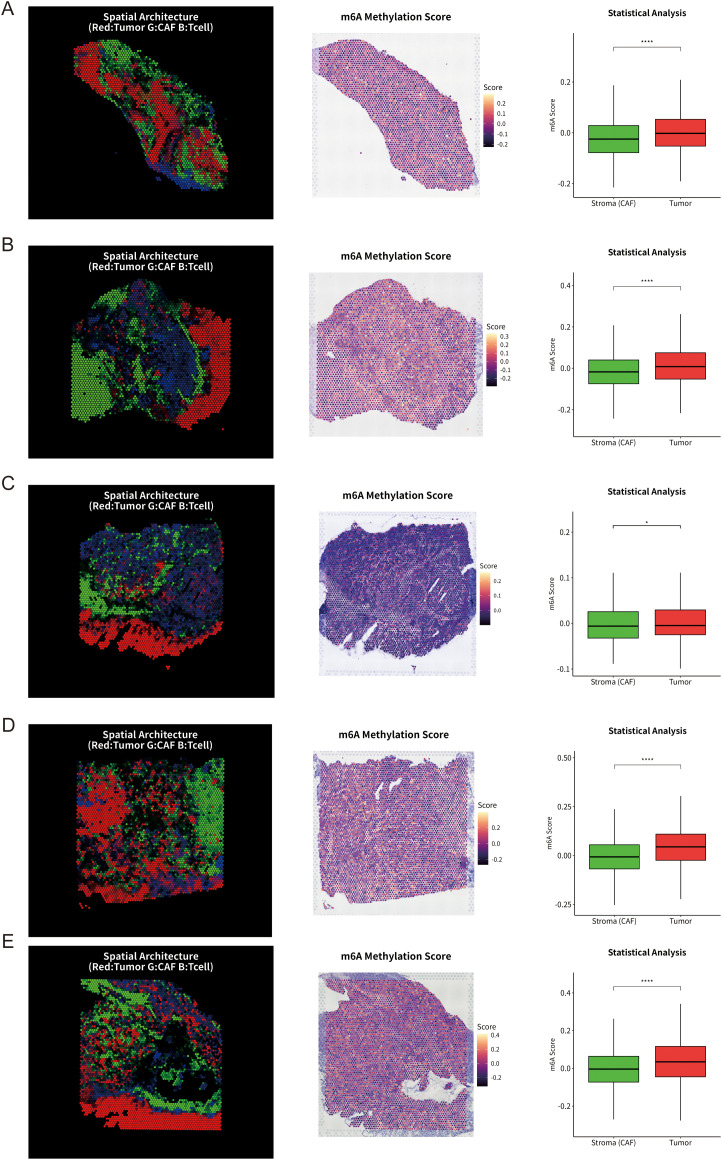
Spatial transcriptomics unveils an m^6^A-associated immune-excluded phenotype characterized by fibroblast-mediated physical barriers. **(A–E)** Five representative Visium spatial transcriptomic sections from gastric cancer tissues. For each section, the left panel shows the reconstructed spatial architecture of the tumor microenvironment, with tumor nests (red), cancer-associated fibroblast (CAF)–rich stroma (green) and T cells (blue) visualized by deconvolution of single-cell–defined cell types. The middle panel displays the corresponding spatial distribution of the composite m^6^A regulator scores calculated from 21 canonical m^6^A regulators using AddModuleScore, with warmer colors indicating higher scores. The right panel presents boxplots comparing m^6^A regulator scores between CAF-rich stroma and tumor nests within each section; statistical significance was determined by the Wilcoxon rank-sum test (*P < 0.05; ****P < 0.0001). Across sections, m^6^A regulator scores are consistently elevated in tumor nests relative to surrounding stroma, indicating a tumor-intrinsic m^6^A program spatially coupled to CAF-mediated encapsulation.

To dissect the spatial heterogeneity of the tumor microenvironment (TME) and identify drivers of immune exclusion, we performed high-resolution spatial transcriptomics (ST) on gastric cancer tissue sections. By integrating single-cell deconvolution with spatial coordinate mapping, we reconstructed the spatial topography of tumor cells, fibroblasts, and T cells. A distinct “immune-excluded” phenotype was strikingly captured in representative samples (**Figures 2A–C**): composite RGB visualization revealed that tumor nests (red) were densely encapsulated by a barrier of fibroblasts (green). This physical obstruction effectively sequestered T cells (blue) within the stroma-rich regions or the invasive margin, preventing their infiltration into the tumor core. Notably, although spatial heterogeneity existed across samples, this characteristic pattern—CAF-mediated tumor encapsulation and restricted T cell infiltration—remained discernible in additional samples (**Figures 2D, E**), confirming the prevalence of this phenotype in gastric cancer.

Given the pivotal role of RNA epitranscriptomics in TME remodeling, we mapped the expression of 21 m^6^A regulators spatially and calculated a site-specific composite m^6^A regulator scores. Spatial overlay analysis revealed a striking concordance between m^6^A modification patterns and histological architecture: high m^6^A regulator scores were specifically enriched within tumor nests, whereas the surrounding stroma manifested as relative “cold spots” with lower scores. Quantitative analysis confirmed that m^6^A regulator scores were significantly upregulated in tumor nests compared to the surrounding CAF-rich stroma. Collectively, these spatial data delineate a unique spatial correlation: tumor-intrinsic upregulation of m^6^A regulators is spatially coupled with the formation of peripheral CAF physical barriers and subsequent T cell exclusion. This suggests that aberrant m^6^A modification may be a key intrinsic driver orchestrating this malignant spatial architecture.

### 3.3 Molecular subtyping based on m^6^A regulators unveils TME heterogeneity and a stroma-driven exclusion mechanism

**Figure 3:**
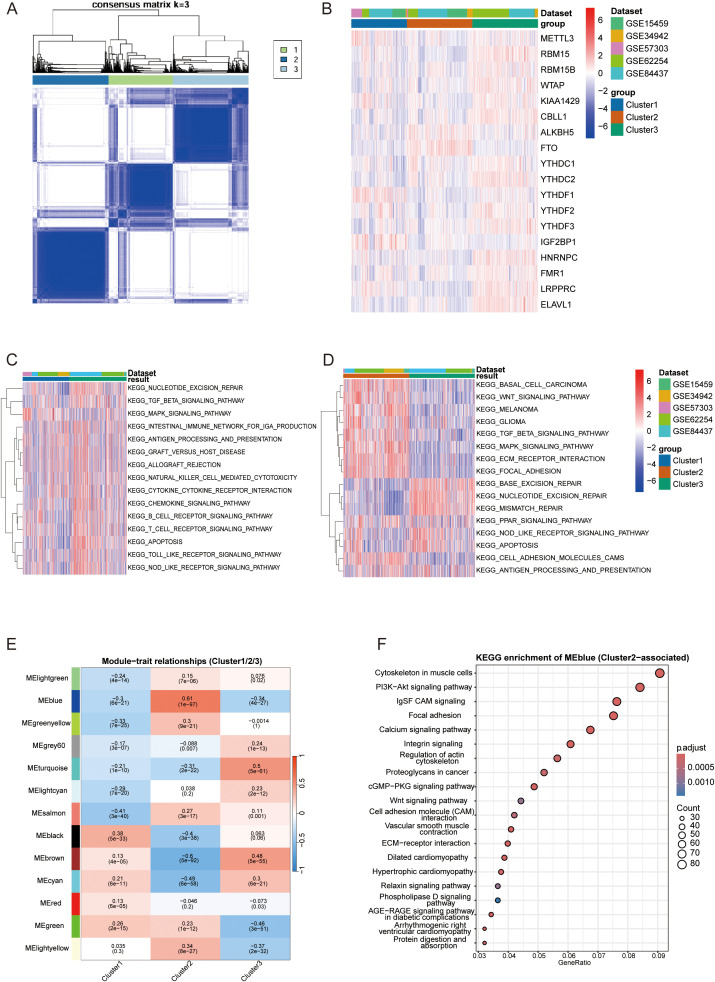
Molecular subtyping based on m^6^A regulators unveils TME heterogeneity and distinct immunological landscapes. **(A)** Consensus matrix heatmap for k = 3 generated by ConsensusClusterPlus based on the expression of 21 canonical m^6^A regulators in the merged GEO cohort, showing a stable partition into three m^6^A clusters. **(B)** Heatmap of m^6^A regulator expression across the three clusters, with samples annotated as Cluster 1–3 on the top bar. **(C)** GSVA heatmap of immune-related KEGG pathways (e.g., T cell receptor signaling, B cell receptor signaling, cytokine–cytokine receptor interaction, antigen processing and presentation), highlighting distinct “desert”, “excluded” and “inflamed” immune phenotypes associated with each m^6^A cluster. **(D)** GSVA-based KEGG pathway enrichment heatmap of stromal- and oncogenic-signaling pathways (e.g., TGF-β signaling, WNT signaling, ECM–receptor interaction, focal adhesion) across the three clusters. **(E)** Heatmap of module–trait relationships between the eigengenes of each co-expression module (rows) and the three m^6^A clusters (columns). Numbers within each cell denote the Pearson correlation coefficient (upper line) and corresponding P value (lower line), highlighting modules most positively correlated with Cluster 2 (immune-excluded phenotype). **(F)** KEGG pathway enrichment analysis for genes within Cluster-2–associated modules, showing significant over-representation of pathways related to focal adhesion, ECM–receptor interaction and Wnt signaling, dot size represents the gene ratio and colour indicates the adjusted P value.

To establish a robust m^6^A-based classification system, we integrated five independent GEO cohorts (GSE15459, GSE34942, GSE57303, GSE62254, GSE84437; total n = 1,050) and extracted the expression matrix of 21 canonical m^6^A regulators after batch correction. Unsupervised consensus clustering identified three molecular subtypes (Clusters 1–3) with high intrinsic stability (**Figure 3A**). Distinct expression patterns of these regulators were visualized in a heatmap (**Figure 3B**), suggesting that specific epigenetic modification modes may drive distinct biological behaviors.

To dissect the immunological landscapes of each m^6^A cluster, we systematically applied GSVA algorithms. Cluster 3 exhibited features of an “immune-inflamed” phenotype, characterized by specific enrichment of adaptive immune pathways (e.g., “T cell receptor signaling pathway”) and innate immune activity (**Figure 3C**). In sharp contrast, Cluster 2 was defined by prominent stromal activation. GSVA analysis revealed that this cluster was specifically enriched in pathways involved in stromal remodeling and cell-matrix interactions, such as “Focal adhesion” and “ECM-receptor interaction” (**Figure 3D**), strongly recapitulating a “stroma-driven immune-excluded” phenotype. Cluster 1 displayed universally low levels of immune and stromal response signatures, consistent with an “immune-desert” phenotype.

To decipher the gene regulatory networks underpinning the immune-excluded phenotype observed in Cluster 2, we further performed Weighted Gene Co-expression Network Analysis (WGCNA).A scale-free network was constructed using an optimized soft-thresholding power (beta=8, scale-free topology fit index > 0.9; **Supplementary Figures 4A, B**.) Module-trait relationship analysis identified the Blue module (MEblue) as the dominant transcriptional driver of Cluster 2, exhibiting the most robust positive correlation (r = 0.61, P = 1e-97; **Figure 3E**). Functional enrichment analysis of hub genes within this module revealed a distinct stromal activation signature. KEGG pathway analysis (**Figure 3F**) demonstrated that pathways critical for extracellular matrix (ECM) dynamics—specifically “PI3K-Akt signaling,” “Wnt signaling pathway”, “Focal adhesion,” and “ECM-receptor interaction”—were significantly overrepresented. The hyperactivation of these pathways within the Blue module suggests that this gene network actively orchestrates profound stromal remodeling and collagen deposition. This mechanism likely constructs a physical barrier that restricts immune cell infiltration, thereby enforcing the immune-excluded state characteristic of Cluster 2.

### 3.4 TIDE analysis delineates TME heterogeneity and predicts divergent responses to immunotherapy across m^6^A phenotypes

**Figure 4:**
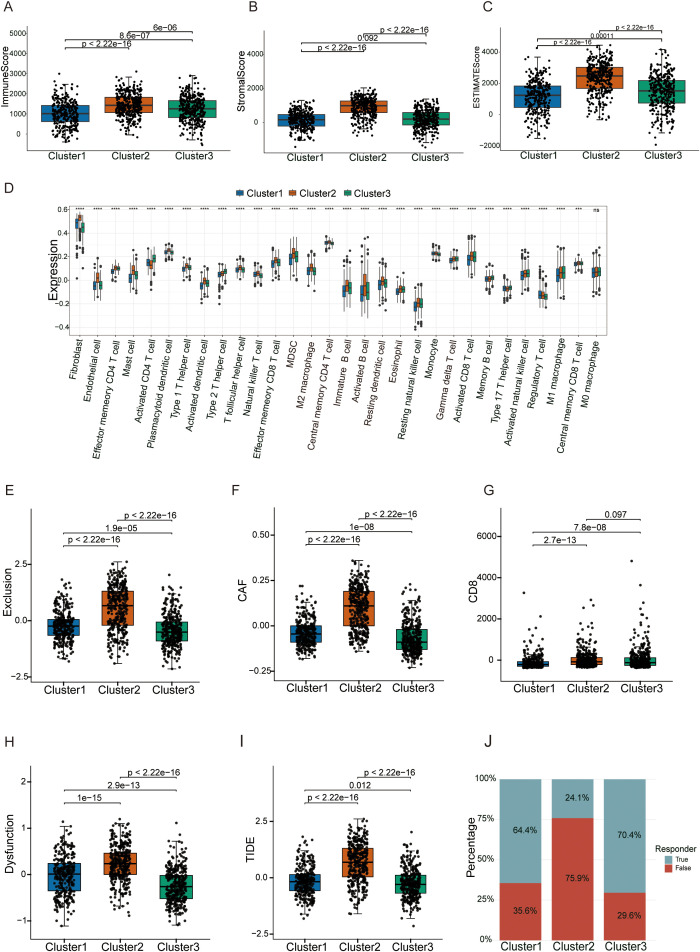
TIDE analysis delineates TME heterogeneity and predicts divergent responses to immunotherapy across phenotypes. **(A–C)** Comparison of ImmuneScore **(A)**, StromalScore **(B)** and ESTIMATEScore **(C)** among the three clusters as calculated by the ESTIMATE algorithm; P values are indicated above the brackets (Wilcoxon rank-sum test). **(D)** Boxplots of ssGSEA scores for representative immune and stromal cell populations (e.g., CD8^+^ T cells, Th1/Th17 cells, NK cells, M1/M2 macrophages, myeloid-derived suppressor cells and cancer-associated fibroblasts) across the three m^6^A clusters. **(E)** T cell–exclusion scores. **(F)** Cancer-associated fibroblast (CAF) scores. **(G)** CD8^+^ T cell relative enrichment scores. **(H)** T cell–dysfunction scores. **(I)** Overall TIDE scores for each cluster, reflecting the combined potential for T cell dysfunction and exclusion; P values for pairwise comparisons among Cluster 1, Cluster 2 and Cluster 3 are indicated above the brackets (Wilcoxon rank-sum test). **(J)** Stacked bar plot showing the proportion of patients predicted to respond or not respond to immune checkpoint blockade (ICB) in each cluster according to the TIDE algorithm.

To elucidate the mechanisms of immune evasion governed by distinct m^6^A patterns, we performed a multidimensional characterization integrating ESTIMATE, ssGSEA, and TIDE algorithms. ESTIMATE analysis provided a global view of the TME landscape: Cluster 3 exhibited quintessential “hot tumor” features, expressed higher ImmuneScore (**Figure 4A**) and ESTIMATEScore (**Figure 4C**). Consistent with this, ssGSEA analysis confirmed robust infiltration of activated immune cells (**Figure 4D**), and this cluster displayed the highest expression of interferon-gamma (IFNG) (**Supplementary Figure 5D**), indicating an active immune state. However, it also displayed significantly elevated expression of CD274 (PD-L1) and Merck18 immunotherapy-response signature scores (**Supplementary Figures 5A, B**), suggesting that adaptive immune resistance via checkpoint upregulation is the primary escape mechanism in this inflamed phenotype.

Cluster 2 presented a paradoxical state of “high infiltration but low function,” it was distinctly defined by the highest StromalScore (**Figure 4B**) and a dramatic enrichment of fibroblasts (**Figure 4D**). TIDE analysis further dissected this phenotype: despite harboring substantial CD8^+^ T cell infiltration (**Figure 4G**), Cluster 2 was characterized by significantly elevated T cell dysfunction scores (**Figure 4H**) and exclusion scores (**Figure 4E**). Mechanistic interrogation identified cancer-associated fibroblasts (CAFs) as the dominant driver: Cluster 2 exhibited CAF scores approximately 1.8 times higher than other clusters (**Figure 4F**). This profound stromal fibrosis likely orchestrates a physical barrier that restricts T cell penetration and induces exhaustion. Notably, while immunosuppressive myeloid cells such as MDSCs and TAMs were also detected (**Supplementary Figures 5C, E**), the dominant stromal signature (**Figures 4B, F**) implies that CAFs are the primary architects of the “immune-excluded” microenvironment in Cluster 2.

Cluster 1, in contrast, displayed universally low ImmuneScores and StromalScores (**Figures 4A, B**), lacking specific immune or stromal response signatures, which fits the definition of an “immune-desert” phenotype.

Predictive Stratification for Immunotherapy Response Leveraging the TIDE scoring system, we evaluated the potential clinical benefit of immune checkpoint inhibitors (ICIs) for each cluster. Cluster 3, with its high PD-L1 expression and lower exclusion signatures, was predicted to derive the greatest benefit from ICI therapy. This was corroborated by the predicted responder analysis, which estimated a response rate of ~70% (**Figure 4J**). Conversely, Cluster 2 exhibited the highest TIDE score (**Figure 4I**) due to severe stromal exclusion and dysfunction, predicting primary resistance to ICIs (responder fraction ~24%; **Figure 4J**). This suggests that ICI monotherapy may be insufficient for these patients, necessitating combination strategies targeting the stromal barrier. Interestingly, despite being an “immune-desert,” Cluster 1 showed a predicted responder fraction comparable to Cluster 3 (**Figure 4J**). This may be attributed to its minimal CAF burden (**Figure 4F**) and the absence of active immunosuppressive mechanisms, rendering the sparse immune cells potentially responsive to reactivation, and this computational estimate should be interpreted cautiously. Collectively, these analyses functionally support the classification of Clusters 1, 2, and 3 as “Desert,” “Excluded,” and “Inflamed” phenotypes, providing a compelling framework for personalized immunotherapy stratification.

### 3.5 Construction and validation of a prognostic risk stratification model based on m^6^A phenotypes

**Figure 5:**
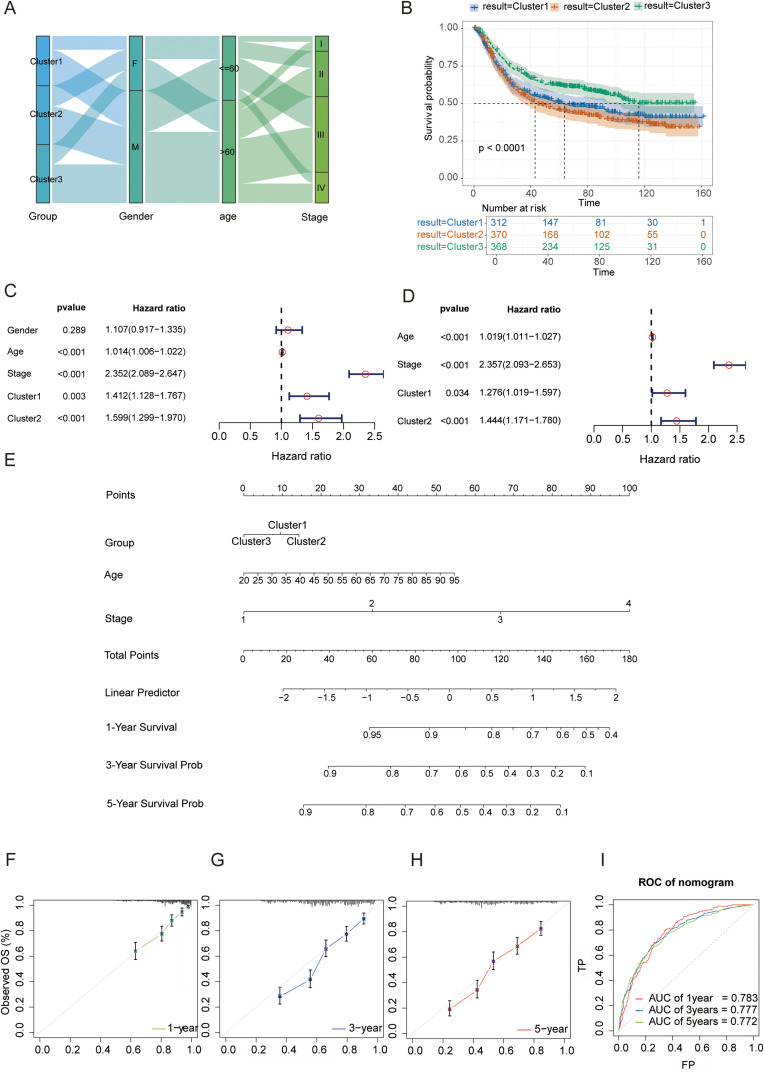
Construction and validation of a prognostic risk stratification model based on m^6^A phenotypes. **(A)** Sankey diagram illustrating the flow distribution of m6A phenotypes across gender, age groups, and clinical stages 2. **(B)** Kaplan–Meier survival curves comparing OS among the three m6A clusters (P < 0.0001, log-rank test). **(C)** Forest plot of univariate Cox regression analyses identifying Age, Clinical Stage, and m6A clusters as significant predictors. **(D)** Forest plot of the optimized multivariate Cox regression analysis (excluding gender), confirming Cluster 2 as a significant independent risk factor (HR = 1.444, P < 0.001). **(E)** Nomogram integrating m6A clusters, age, and clinical stage for predicting individualized OS. **(F–H)** Calibration plots at 1 years **(F)**, 3 year **(G)**, and 5 years **(H)** showing the accuracy of the nomogram. **(I)** Time-dependent ROC curves of the nomogram for 1-, 3-, and 5-year OS, with corresponding AUC values indicated.

To deconstruct the clinical relevance of phenotypes, we first utilized a Sankey diagram (**Figure 5A**) to map the flow distribution between molecular clusters and clinicopathological features. This visualization revealed a distinct enrichment of the immune-excluded phenotype (Cluster 2) in patients with advanced gastric cancer (Stage III–IV), suggesting its intrinsic link to tumor progression. Kaplan–Meier survival analysis (**Figure 5B**) confirmed that patients with Cluster 2 exhibited significantly worse overall survival (P < 0.0001) compared to the immune-inflamed Cluster 3, while Cluster 1 showed an intermediate prognosis.

To identify independent prognostic drivers, we performed univariate and multivariate Cox proportional hazards regression analyses. Univariate analysis (**Figure 5C**) indicated that m6A phenotypes, age, and clinical stage were significantly associated with mortality risk (P < 0.05), while gender was not a significant predictor (P = 0.289). In the optimized multivariate model (**Figure 5D**), after excluding the non-significant factor of gender, Cluster 2 was confirmed as a robust independent risk factor (HR = 1.444, 95% CI: 1.171–1.780, P < 0.001).

Leveraging these validated predictors, we constructed a nomogram (**Figure 5E**) integrating m6A cluster, age, and stage for individualized OS estimation. The model exhibited high predictive fidelity, as demonstrated by the calibration curves (**Figures 5F–H**), which showed excellent agreement between predicted and actual survival at 1, 3, and 5 years. Furthermore, time-dependent ROC analysis (**Figure 5I**) confirmed the system’s robust predictive efficacy, with AUC values of 0.783, 0.777, and 0.772 for 1-, 3-, and 5-year OS, respectively.

### 3.6 Chemotherapy drug sensitivity analysis

We employed transcriptome-based IC50 predictions and compared chemotherapy and targeted drug responses across three m^6^A phenotypes using the Genomic Drug Sensitivity Consortium (GDSC) resource (**Supplementary Figure 6**). IC50 values for 5-fluorouracil, docetaxel, paclitaxel, gemcitabine, and lapatinib were significantly elevated in the exclusion phenotype (all pairwise differences significant; exact P-values indicated in figure). For EGFR-targeted agents cetuximab and erlotinib, inter-cluster differences were observed but of lesser magnitude. Differences for cisplatin were non-significant (P = 0.53). Collectively, these predictive data reveal a broad chemoresistance bias in immune-rejecting tumors.

### 3.7 Histopathological validation: FMR1 expression is spatially coupled with the immune-excluded phenotype

**Figure 6:**
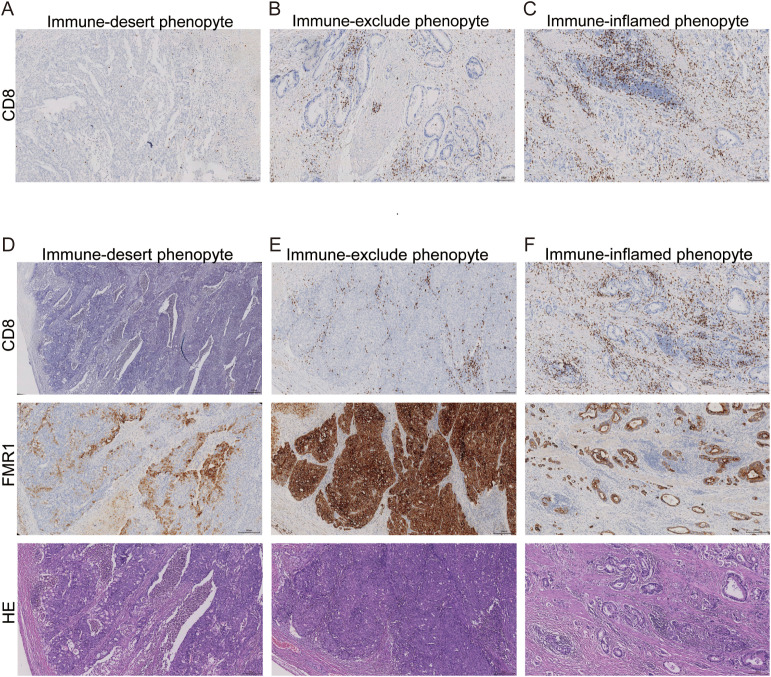
Immunohistochemical staining reveals high FMR1 expression in the immune-exclude phenotype. **(A–C)** Representative CD8 immunohistochemical staining of gastric cancer specimens illustrating three spatial immune phenotypes: immune-desert **(A)**, with a near-complete absence of CD8^+^ T cells in both tumor core and stroma; immune-excluded **(B)**, with dense CD8^+^ T cell accumulation at the tumor–stroma interface and invasive margin but minimal intratumoral infiltration; and immune-inflamed **(C)**, with abundant CD8^+^ T cells diffusely infiltrating tumor nests and surrounding stroma. **(D–F)** Serial sections of representative cases further illustrating CD8, FMR1 and H&E staining patterns in immune-desert **(D)**, immune-excluded **(E)** and immune-inflamed **(F)** phenotypes. In immune-excluded tumors, strong and diffuse FMR1 staining is observed in tumor cells and spatially coincides with peritumoral CD8^+^ T cell arrest, whereas FMR1 expression is weaker or more heterogeneous in immune-desert and immune-inflamed samples. Scale bars, 200 μm.

To validate our transcriptomic classification at the histopathological level, we performed systematic immune phenotyping of gastric cancer specimens using CD8 immunohistochemistry (IHC). Based on the spatial distribution patterns of CD8^+^ T cells relative to tumor nests, specimens were stratified into three distinct phenotypes (**Figures 6A–C**): Immune-desert: Characterized by a profound paucity of CD8^+^ T cell infiltration in both the tumor core and stroma. Immune-excluded: Defined by the dense accumulation of CD8^+^ T cells in the stroma or invasive margins, which are physically restricted from penetrating the tumor parenchyma, reflecting a “ excluded” status. Immune-inflamed: Exhibiting diffuse and abundant infiltration of CD8^+^ T cells throughout the tumor nests and surrounding stroma.

Subsequently, utilizing serial sectioning techniques, we spatially aligned CD8 staining, FMR1 expression, and histological architecture (H&E) to decode the relationship between FMR1 levels and immune topography (**Figures 6D–F**). Comparative analysis revealed a striking concordance: regions classified as “immune-excluded” exhibited diffuse, strong positive staining for FMR1 within tumor cells. This FMR1 upregulation spatially coincided with the peritumoral arrest of CD8^+^ T cells. In contrast, FMR1 expression was comparatively attenuated or sporadic in immune-inflamed and immune-desert phenotypes. Semi-quantitative statistical analysis of the cohort corroborated these observations (**Table 2**). Frequency distribution analysis demonstrated a significant positive correlation between FMR1 expression intensity and the immune-excluded phenotype (P < 0.05). This clinicopathological evidence robustly establishes aberrant FMR1 accumulation as a hallmark molecular feature of the immune-excluded microenvironment, reinforcing the hypothesis that FMR1 drives T cell exclusion.

**Table 2:** Correlation between FMR1 expression level and tumor immunophenotype in gastric cancer.

Immune phenotype	n	FMR1 - + ++ +++				P value	
Immune desert	20	3	7	6	4	P>0.05	
Immune exclude	32	3	4	16	9	P<0.05	P<0.05
Immune inflamed	18	5	4	6	3	P>0.05	

“−” indicates negative; “+” indicates weakly positive; “++” indicates moderately positive; “+++” indicates strongly positive.

### 3.8 Multiplex immunofluorescence reveals high FMR1 expression in the immune-excluded phenotype

**Figure 7:**
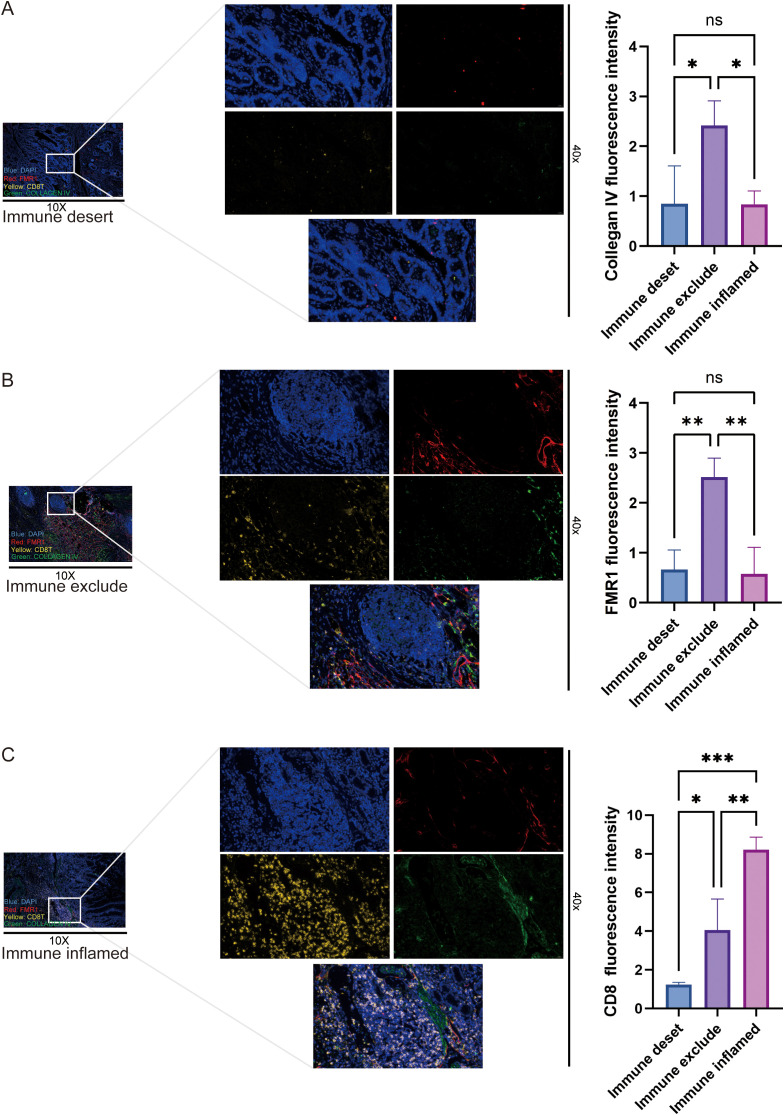
Histopathological validation: FMR1 expression is spatially coupled with the immune-excluded phenotype. **(A–C)** Representative multiplex immunofluorescence images of gastric cancer tissues showing immune-desert **(A)**, immune-excluded **(B)** and immune-inflamed **(C)** phenotypes. Low-magnification views (10×) are shown on the left and the corresponding high-magnification fields (40×) on the right. Nuclei are stained with DAPI (blue), CD8 marks cytotoxic T cells (yellow), collagen IV delineates the stromal barrier (green), and FMR1 is shown in red. In immune-excluded samples, CD8^+^ T cells predominantly accumulate within collagen IV–rich peritumoral stroma and fail to penetrate the tumor nests, which display strong FMR1 expression. In immune-inflamed samples, CD8^+^ T cells extensively infiltrate the tumor epithelium and stroma, accompanied by relatively weak FMR1 signal. In immune-desert samples, CD8^+^ T cells are largely absent and only scattered FMR1-positive cells are observed. Quantitative comparison of fluorescence intensities for FMR1, CD8^+^ T cells and collagen IV among the three immune phenotypes shows that FMR1 expression is significantly higher in the immune-excluded phenotype than in the immune-inflamed and immune-desert phenotypes, whereas the latter two do not differ significantly. Statistical significance was determined by one-way ANOVA. **P < 0.01$, ***P < 0.001, ****P < 0.0001; ns, not significant.

Using multiplex immunofluorescence staining, nuclei were labeled with DAPI (blue), cytotoxic T cells were marked with CD8 (yellow), and the stromal barrier was indicated by collagen IV (green). FMR1 was represented in red. Based on this, gastric cancer tissues were classified into immune-excluded, immune inflamed, and immune-desert phenotypes (**Figures 7A–C**). In immuno-exclude samples, CD8^+^ T cells were mainly aggregated in the peritumoral stromal area, partially overlapping with collagen IV deposition, while FMR1 showed strong positive expression. In the inflamed phenotype, abundant CD8^+^ T cells infiltrated deeply into the tumor epithelium, and the FMR1 fluorescence signal was weak. In the immune-desert phenotype, CD8^+^ T cells were nearly absent, with only scattered cells observed. Quantitative analysis of FMR1 fluorescence intensity across different immune phenotypes revealed that its expression level was significantly higher in the immuno-exclude type than in the other two types, while no significant difference was found between the inflamed and desert types. This suggests that high FMR1 expression is closely associated with defective T-cell infiltration or an excluded tumor microenvironment, which is consistent with the immunohistochemistry results.

### 3.9 FMR1 associates with FTO and is linked to FTO protein turnover through an MG132-sensitive pathway

**Figure 8:**
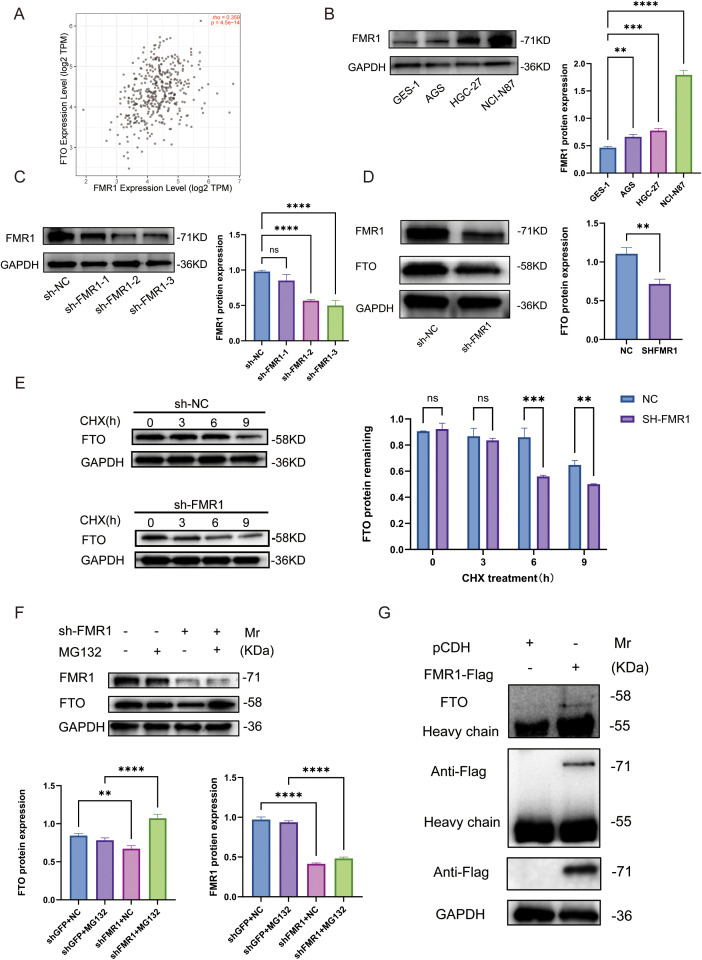
FMR1 associates with FTO and is linked to FTO protein turnover through an MG132-sensitive pathway. **(A)** Scatter plot showing the positive correlation between FMR1 and FTO mRNA expression in gastric cancer samples from the GEPIA database (Spearman’s r and P value shown in the upper-right corner). **(B)** Western blot and quantification of FMR1 protein levels in normal gastric epithelial cells (GES-1) and gastric cancer cell lines (AGS, HGC-27 and NCI-N87). **(C)** Western blot validation of FMR1 knockdown efficiency in NCI-N87 cells transduced with three independent shRNAs (sh-FMR1-1/2/3) compared with sh-NC control, with densitometric analysis on the right. **(D)** Western blot showing that FMR1 silencing reduces FTO protein expression, with quantification of FTO levels normalized to GAPDH. **(E)** Cycloheximide (CHX) chase assay assessing FTO protein stability in sh-NC and sh-FMR1 NCI-N87 cells treated with CHX (100 μg/mL) for the indicated times (0, 3, 6, 9 h); right panel shows relative FTO levels over time, demonstrating a shortened half-life upon FMR1 knockdown. **(F)** Western blot analysis of FTO and FMR1 protein levels in sh-NC and sh-FMR1 cells treated with or without the proteasome inhibitor MG132 (10 μM, 6 h); bar graphs show that MG132 rescues the FTO reduction induced by FMR1 knockdown, implicating the ubiquitin–proteasome pathway in FTO degradation. **(G)** Co-immunoprecipitation of Flag-tagged FMR1 in NCI-N87 cells using anti-Flag beads followed by immunoblotting for endogenous FTO, confirming a physical interaction between FMR1 and FTO at the protein level; input controls are shown in the lower panel. Data in bar graphs are presented as mean ± SD; **P < 0.01, ***P < 0.001, ****P < 0.0001; ns, not significant.

Correlation analysis using the GEPIA database revealed a significant positive correlation between *FMR1* and *FTO* mRNA levels in gastric cancer tissues (R = 0.359, P < 0.0001), suggesting a co-regulatory network (**Figure 8A**). Western blotting further verified that FMR1 protein expression was significantly upregulated in multiple gastric cancer cell lines (AGS, HGC-27, NCI-N87) compared to normal gastric epithelial cells (GES-1) (**Figure 8B**), implying a potential oncogenic role. To validate the regulatory effect of FMR1 on FTO, we established stable FMR1-knockdown cell lines using shRNA (**Figure 8C**). Notably, FMR1 silencing resulted in a significant downregulation of FTO protein levels compared to controls (**Figure 8D**). Cycloheximide (CHX) chase assays elucidated the mechanism underlying this downregulation: the half-life of FTO protein was markedly shortened in FMR1-knockdown cells, indicating that FMR1 contributes to maintaining FTO protein stability (**Figure 8E**).

To delineate the specific degradation pathway responsible for the increased FTO instability, we performed rescue assays using the proteasome inhibitor MG132. The results demonstrated that MG132 treatment effectively reversed the reduction in FTO protein levels induced by FMR1 knockdown (**Figure 8F**), supporting that FMR1 protects FTO from degradation via a proteasome dependent mechanism. However, ubiquitination of FTO was not directly assessed in this study. Finally, co-immunoprecipitation (Co-IP) assays provided supporting biochemical evidence Flag-tagged FMR1 specifically pulled down endogenous FTO protein(**Figure 8G**). Molecular docking and molecular dynamics simulations are provided as supportive evidence for structural compatibility and complex stability (**Supplementary Figures S7**, **S8**).

Notably, because FMR1 is an RNA-binding protein, the observed co-immunoprecipitation indicates association within a complex; RNase-controlled and reciprocal endogenous Co-IP experiments will be required to establish whether the interaction is direct and RNA-independent. Collectively, these data suggest a model in which FMR1 is linked to the maintenance of FTO protein abundance and turnover in gastric cancer cells, consistent with a post-translational regulatory mechanism.

## 4 Discussion

The high heterogeneity of gastric cancer and its complex tumor microenvironment (TME) are core factors contributing to the low clinical predicted responder fraction of immune checkpoint inhibitors (ICIs). In this study, we systematically identified an “immune-excluded” gastric cancer phenotype characterized by excessive activation of the FMR1-FTO axis by integrating single-cell sequencing, spatial transcriptomics, and multi-cohort bulk transcriptomic data. Our work not only reveals the intrinsic link between m^6^A modification patterns and physical barriers to CD8+ T cells in the spatial dimension but also achieves a breakthrough in the previously unknown non-canonical functional domain of FMR1. Our data suggest a potential post-translational mechanism by which FMR1 may stabilize FTO protein, thereby contributing to an immunosuppressive microenvironment.

Previous studies predominantly focused on the effects of m^6^A regulators on tumor cell-intrinsic proliferation (40), overlooking their role in reshaping the spatial architecture of the TME. Our spatial transcriptomics data visually demonstrate the anatomical essence of “immune exclusion”: high m^6^A regulator scores are specifically confined to tumor nests, precisely the region enveloped by a dense CAF barrier, forcing CD8^+^ T cells to remain trapped in the stroma. Importantly, this “m6A regulator score” reflects the coordinated expression pattern of canonical m^6^A writers, erasers and readers, rather than a direct measurement of absolute global m^6^A abundance. Thus, it captures an activated epitranscriptomic regulatory program at the network level, in which upregulated writers and readers can coexist with increased FTO expression. In such a context, FTO is unlikely to erase m^6^A marks indiscriminately across the transcriptome; instead, it is more plausible that FTO acts in a substrate- and context-dependent manner on a restricted subset of transcripts, while the majority of m^6^A events driven by heightened writer activity remain intact or even increased. This framework reconciles the apparent conceptual paradox of a “high m^6^A regulator signature” co-occurring with elevated expression of the demethylase FTO.

The Cluster 2 phenotype, identified through unsupervised clustering, closely recapitulates the spatially defined immune-excluded architecture, exhibiting the highest stromal score and T cell dysfunction score. This finding resonates with Mariathasan et al.’s concept of “TGF-β-driven immune exclusion” in urothelial carcinoma (41), but extends it by implicating an m^6^A regulator program as an upstream epitranscriptomic layer associated with this phenotype. TIDE analysis further revealed that Cluster 2 exhibited the poorest predicted response to ICIs, suggesting that PD-1/PD-L1 antibodies alone may be insufficient for these patients. Instead, disrupting the physical barrier formed by CAFs or modulating m^6^A-related signaling modules may be required as a prerequisite to restore effective T cell infiltration.

The most significant innovation of this study lies in revealing an unconventional mechanism by which FMR1 regulates FTO stability in gastric cancer. FMR1 is typically recognised as an RNA-binding protein involved in mRNA transport, stability and translational control (42). Importantly, Our biochemical experiments, including Co-IP, demonstrate an association between FMR1 and FTO and suggest that FMR1 may attenuate proteasome-mediated degradation of FTO. This observation broadens the traditional view of FMR1 as a purely RNA-centred regulator and suggests that FMR1 may also function as a molecular chaperone or scaffold that reduces FTO turnover and maintains a high intracellular pool of FTO protein in a proteasome-dependent manner.

Within the globally activated m^6^A regulator landscape observed in immune-excluded tumors, FTO is therefore best viewed as a selective “fine-tuner” rather than a global eraser. Stabilized by FMR1, FTO is poised to demethylate a subset of transcripts that are particularly relevant to stromal remodelling and immune evasion, instead of uniformly reducing m^6^A levels across the transcriptome. As the primary m^6^A demethylase, elevated FTO expression is likely to alter mRNA modification states on key immune activation and stromal remodelling genes, potentially forming the molecular basis for inducing CAF activation and collagen deposition (43–45). Although this study has not yet fully delineated specific downstream targets of FTO in this context, Yang et al. previously reported that FTO promotes PD-L1 mRNA degradation through demethylation (46, 47). However, PD-L1 expression was not elevated in our exclude-type (Cluster 2) group, suggesting that, in the setting of gastric cancer immune exclude, FTO may preferentially act on alternative substrates (e.g. components of pro-fibrotic TGF-β or ECM pathways), which warrants further investigation. Future work incorporating global and transcript-specific m^6^A profiling will be essential to clarify how the FMR1–FTO module reshapes the epitranscriptome at the level of individual targets.

Notably, in the initial survival analyses, the immune-excluded phenotype (Cluster 2) was associated with markedly worse overall survival in univariate models, yet the hazard ratio appeared attenuated or inverted after multivariable adjustment. We interpret this pattern not as evidence that the immune-excluded state is “protective,” but rather as a statistical consequence of strong collinearity with advanced clinical stage (Stage III/IV), which is also evident from the clinical-feature flow in the Sankey diagram. In this context, the excess mortality observed in Cluster 2 is largely attributable to the high prevalence of late-stage disease at diagnosis, accompanied by prominent stromal fibrosis.

Importantly, after refining the multivariable model by excluding the non-informative covariate (gender), Cluster 2 remained a significant independent adverse prognostic factor (HR = 1.444, 95% CI: 1.171–1.780, P < 0.001), supporting the robustness of the association beyond conventional clinicopathological variables. Together, these findings are consistent with the hypothesis that an immune-excluded microenvironment may actively facilitate local invasion and metastatic dissemination—thereby enriching for advanced-stage presentation—rather than merely reflecting staging differences. Accordingly, incorporating m^6^A-based clustering into prognostic assessment may provide biologically informative risk stratification that complements, and potentially refines, TNM staging.

Although this study provides multi-level evidence, certain limitations remain. First, our conclusions regarding the role of the FMR1–FTO axis in immune exclusion are primarily based on correlative analysis of clinical samples and *in vitro* mechanistic validation using cell lines, lacking *in vivo* causal evidence from immunocompetent mouse models and functional interrogation of T cell behaviour. Second, due to the resolution limitations of current spatial transcriptomics technologies, we were unable to precisely resolve the subcellular distribution of FMR1 and FTO at the CAF–tumor cell interface. Third, we inferred an activated m^6^A regulator program from expression data rather than directly quantifying global or transcript-specific m^6^A abundance; dedicated epitranscriptomic assays will be required to substantiate the proposed model of FMR1–FTO-mediated m^6^A reprogramming. Furthermore, the bias observed in Cluster 2 during multivariate analysis suggests that larger, prospectively collected cohorts are needed to validate the independent prognostic value and therapeutic relevance of this classification. While our data support an association between FMR1 and FTO and suggest MG132-sensitive regulation of FTO protein turnover, several mechanistic gaps remain. We did not perform RNase-treated or reciprocal endogenous Co-IP assays to determine whether the FMR1–FTO association is RNA-independent and direct. In addition, FTO ubiquitination and rescue experiments (re-expression of FTO after FMR1 knockdown) were not conducted, precluding definitive causal closure. Finally, FTO mRNA levels under FMR1 perturbation were not quantified, and thus transcriptional contributions cannot be fully excluded. Future work using these assays, together with *in vivo* models, will be essential to validate the proposed post-translational mechanism and its functional relevance to immune exclusion.

## Data Availability

Publicly available datasets were analyzed in this study. This data can be found here: All datasets analyzed in this study are publicly available from open-access databases, as detailed in the Materials and Methods section. Specifically, bulk RNA-seq and single-cell RNA-seq data were obtained from the Gene Expression Omnibus (GEO) under accession numbers provided in the article.
