# Supplementary material for: Integrative single-cell, spatial, and bulk transcriptomics reveal an FMR1–FTO axis linked to the immune-excluded phenotype in gastric cancer
Source: Front Immunol. 2026 Mar 9;17:1713267. doi: 10.3389/fimmu.2026.1713267 (PMC13006672; doi:10.3389/fimmu.2026.1713267)
Supplement: Supplementary file 1 [file Table1.docx]

**Supplementary materials**

**Supplementary Table 1** ssGSEA-based estimation of immune cell and stromal cell infiltration in gastric cancer samples.

| Activated B cell |
| --- |
| Immature B cell |
| Activated CD4 T cell |
| Activated CD8 T cell |
| Type 1 T helper cell |
| Type 2 T helper cell |
| Type 17 T helper cell |
| Effector memeory CD4 T cell |
| Effector memeory CD8 T cell |
| Gamma delta T cell |
| Central memory CD8 T cell |
| Central memory CD4 T cell |
| T follicular helper cell |
| Memory B cell |
| Regulatory T cell |
| Activated natural killer cell |
| Resting natural killer cell |
| Monocyte |
| Eosinophil |
| Resting dendritic cell |
| Activated dendritic cell |
| Plasmacytoid dendritic cell |
| M0 macrophage |
| M1 macrophage |
| M2 macrophage |
| MDSC |
| Neutrophil |
| Mast cell |
| Natural killer T cell |
| Endothelial cell |
| Fibroblast |

**
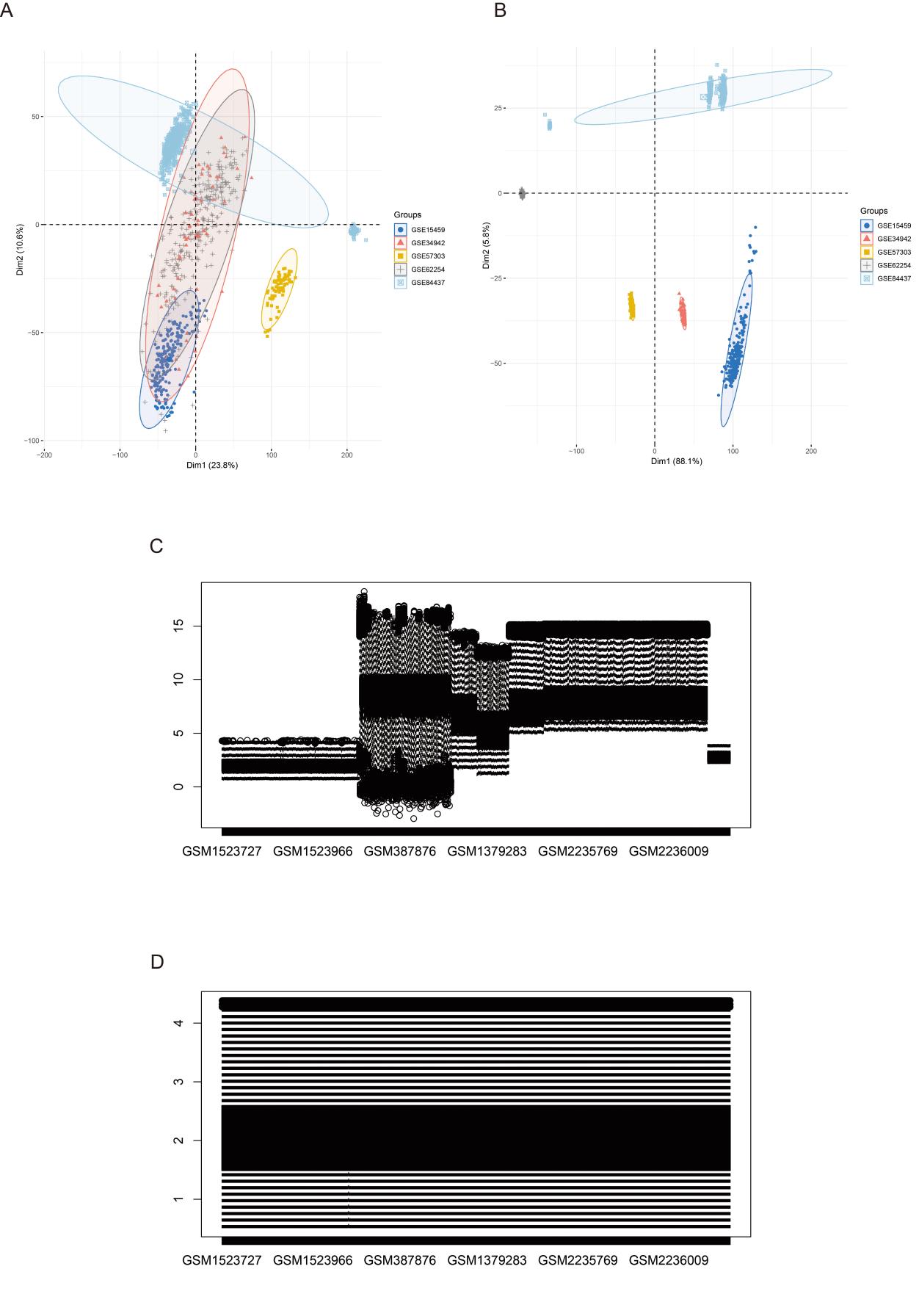
**

**Supplementary Figure S1.** Batch-effect correction across bulk cohorts: PCA before and after harmonization with accompanying distribution diagnostics.

**
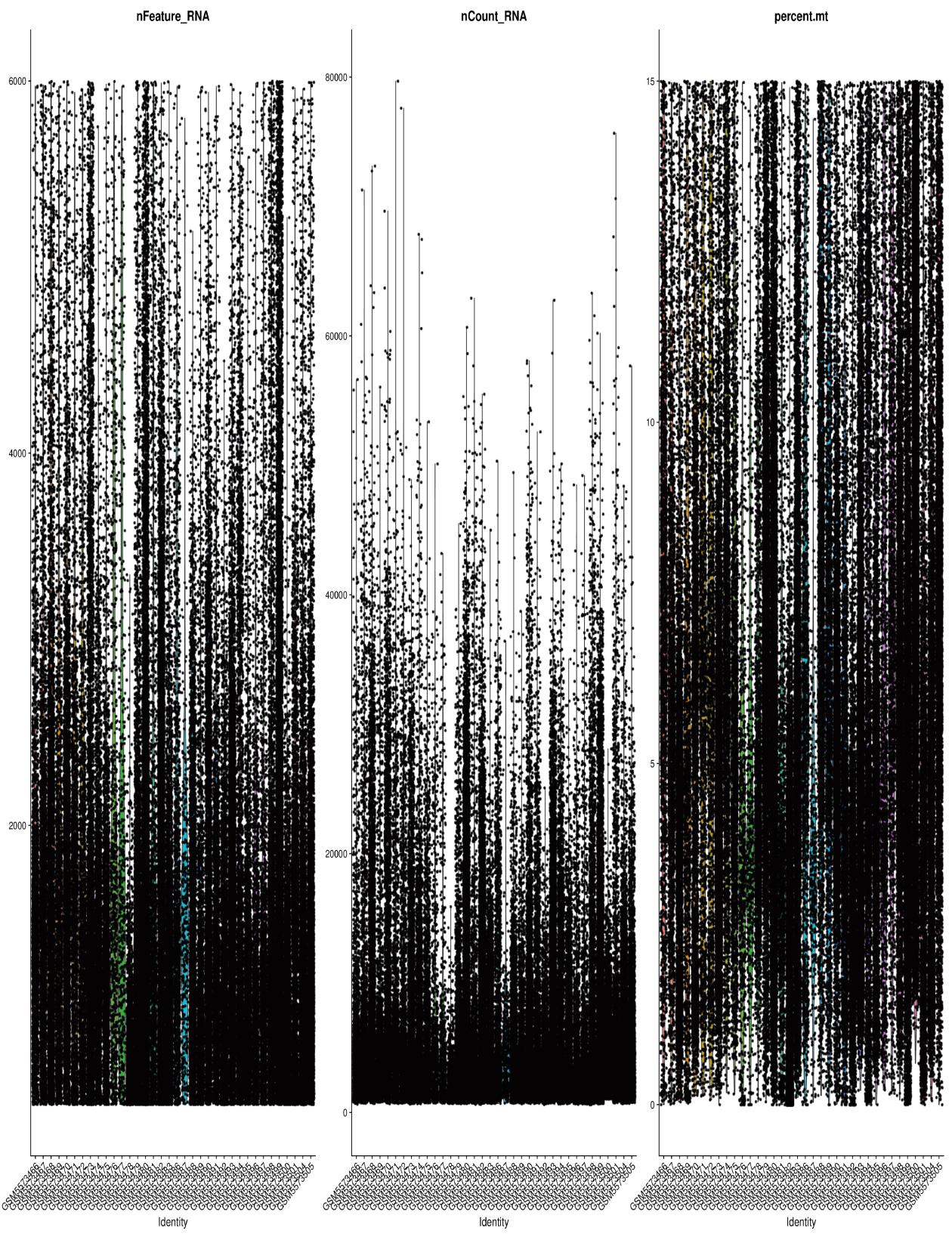
**

**Supplementary Figure S2.** Single-cell quality control (Seurat): distributions of nFeature_RNA, nCount_RNA, and mitochondrial percentage across cells/samples.


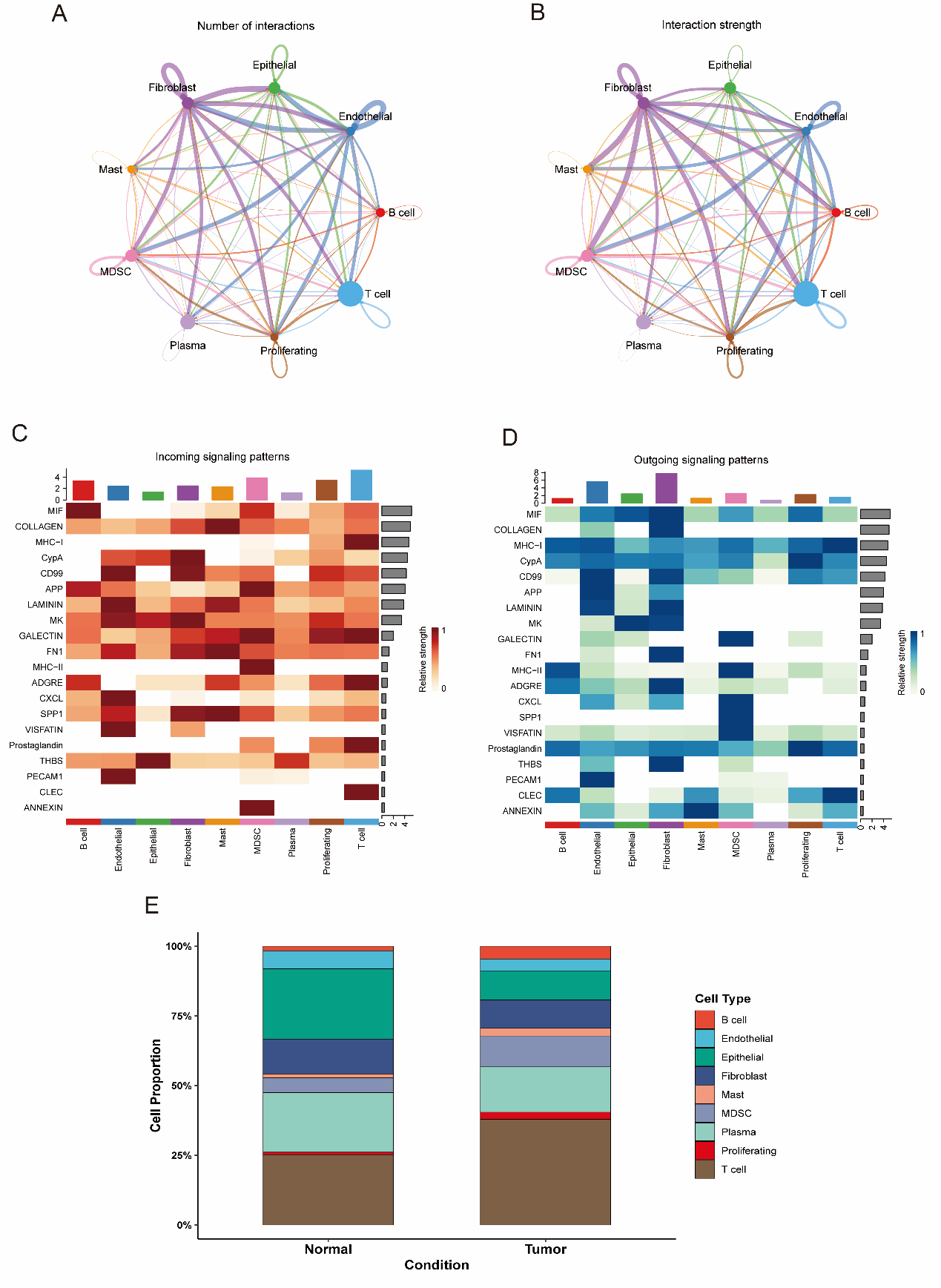


**Supplementary Fig. S3. Single-cell communication and cellular composition analyses reveal fibroblast-centered signaling networks in gastric cancer.** **(A)** Circle plot showing the number of inferred ligand–receptor interactions among the nine major cell types, with edge width proportional to interaction counts and node size reflecting overall connectivity. (B) Circle plot depicting aggregate interaction strength between cell types, with thicker edges indicating stronger communication signals. (C) Heatmap of incoming signaling patterns across cell types inferred by CellChat; rows represent signaling pathways and columns indicate receiver cell types, with color intensity denoting relative signaling strength. (D) Heatmap of outgoing signaling patterns across cell types; rows represent pathways and columns indicate sender cell types, highlighting major signal-producing populations. (E) Stacked bar plot comparing cell-type composition between normal and tumor tissues, showing proportional shifts in major lineages associated with malignant transformation.


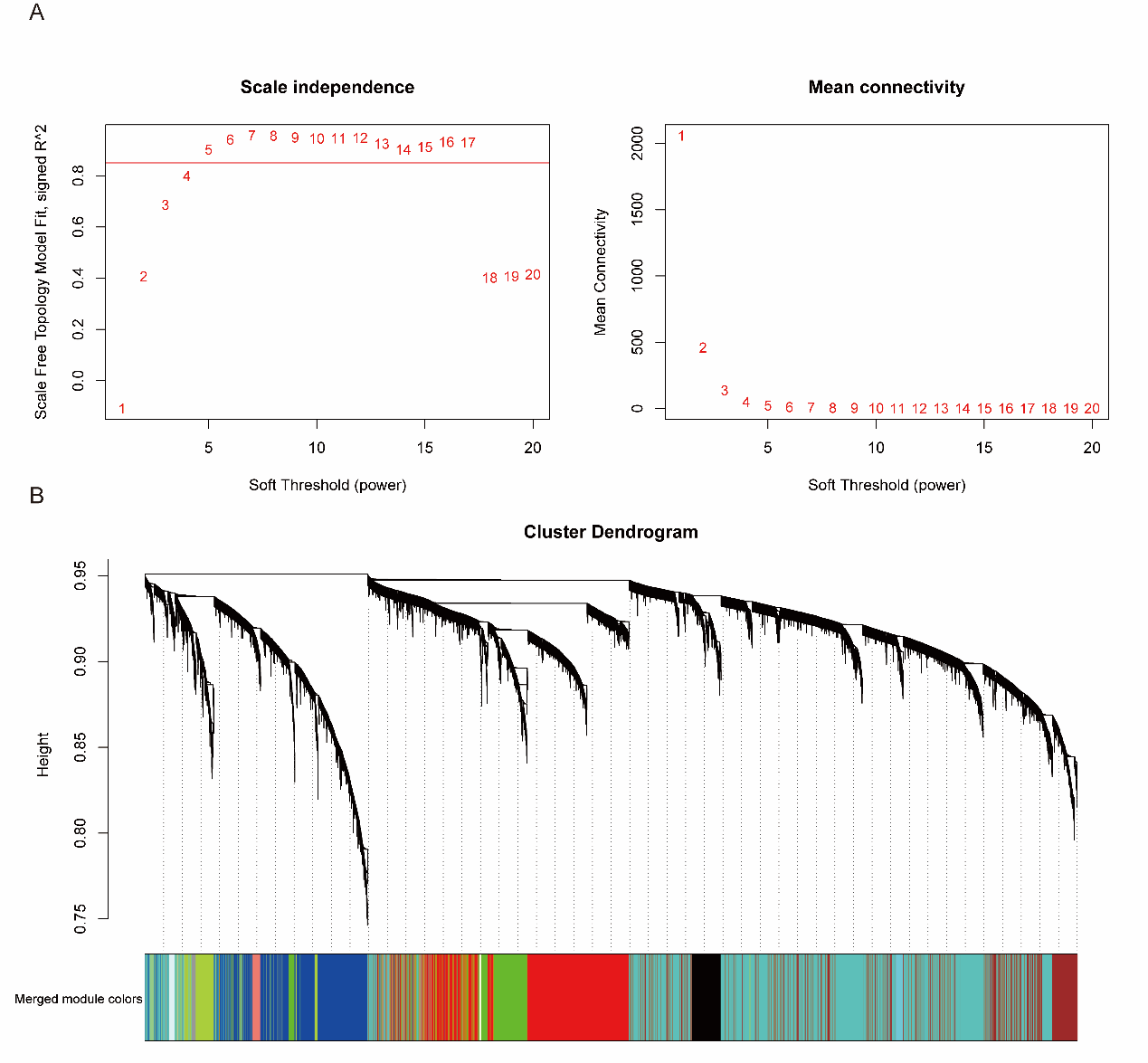


**Supplementary Fig. S4. WGCNA network construction and module identification in the merged gastric cancer cohort.**

(A) Determination of the soft-thresholding power (β) for scale-free topology. The left panel shows the scale-free topology fit index (signed R²) across a range of candidate powers, with the red line indicating the target threshold for approximate scale-free topology. The right panel depicts the corresponding mean connectivity, illustrating the trade-off between network fit and connectivity as β increases. The optimal β was selected based on achieving a high scale-free fit while maintaining adequate connectivity.

(B) Hierarchical clustering dendrogram of genes based on topological overlap, with the color bar indicating the dynamically detected and subsequently merged co-expression modules. Each branch represents a gene cluster, and modules are defined as groups of highly co-expressed genes used for downstream module trait correlation and functional enrichment analyses.


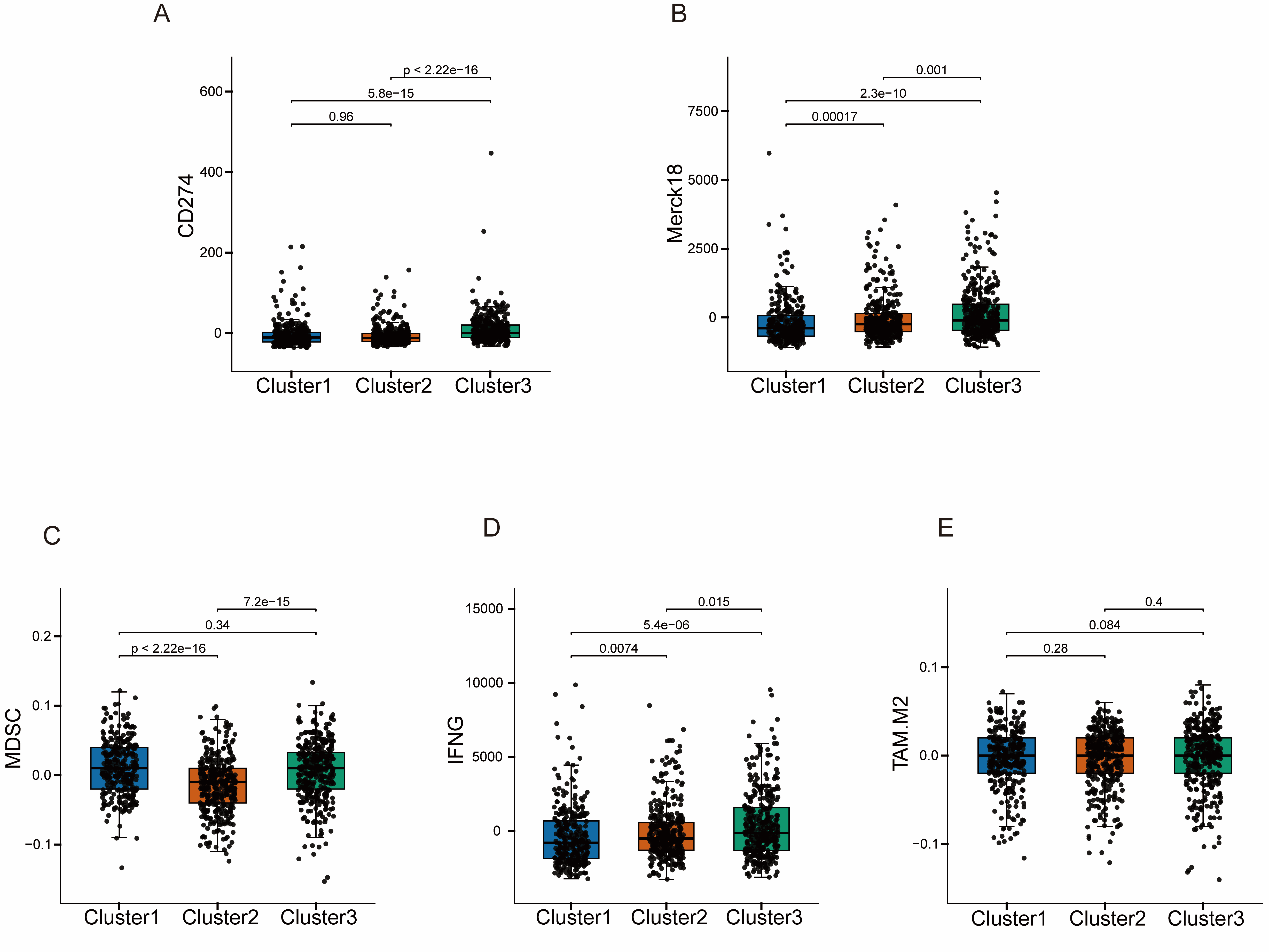


**Supplementary Fig. S5. Additional TIDE-associated immune metrics across m⁶A-based clusters.** (A) Comparison of CD274 (PD-L1) expression among Cluster 1–3, with pairwise P values indicated. (B) Distribution of the Merck18 immunotherapy-response signature score across clusters, reflecting differential predicted ICI benefit. (C) Comparison of MDSC enrichment scores among clusters, highlighting differences in myeloid-driven immunosuppression. (D) Differences in IFNG-related signal/activity (IFNG metric) across clusters, indicating variation in T-cell inflammatory programs. (E) Comparison of TAM.M2 (M2-like tumor-associated macrophage) enrichment scores among clusters, summarizing macrophage-associated immunosuppressive components. Box plots show median and interquartile range; points represent individual samples, and pairwise statistical significance is annotated as P values.


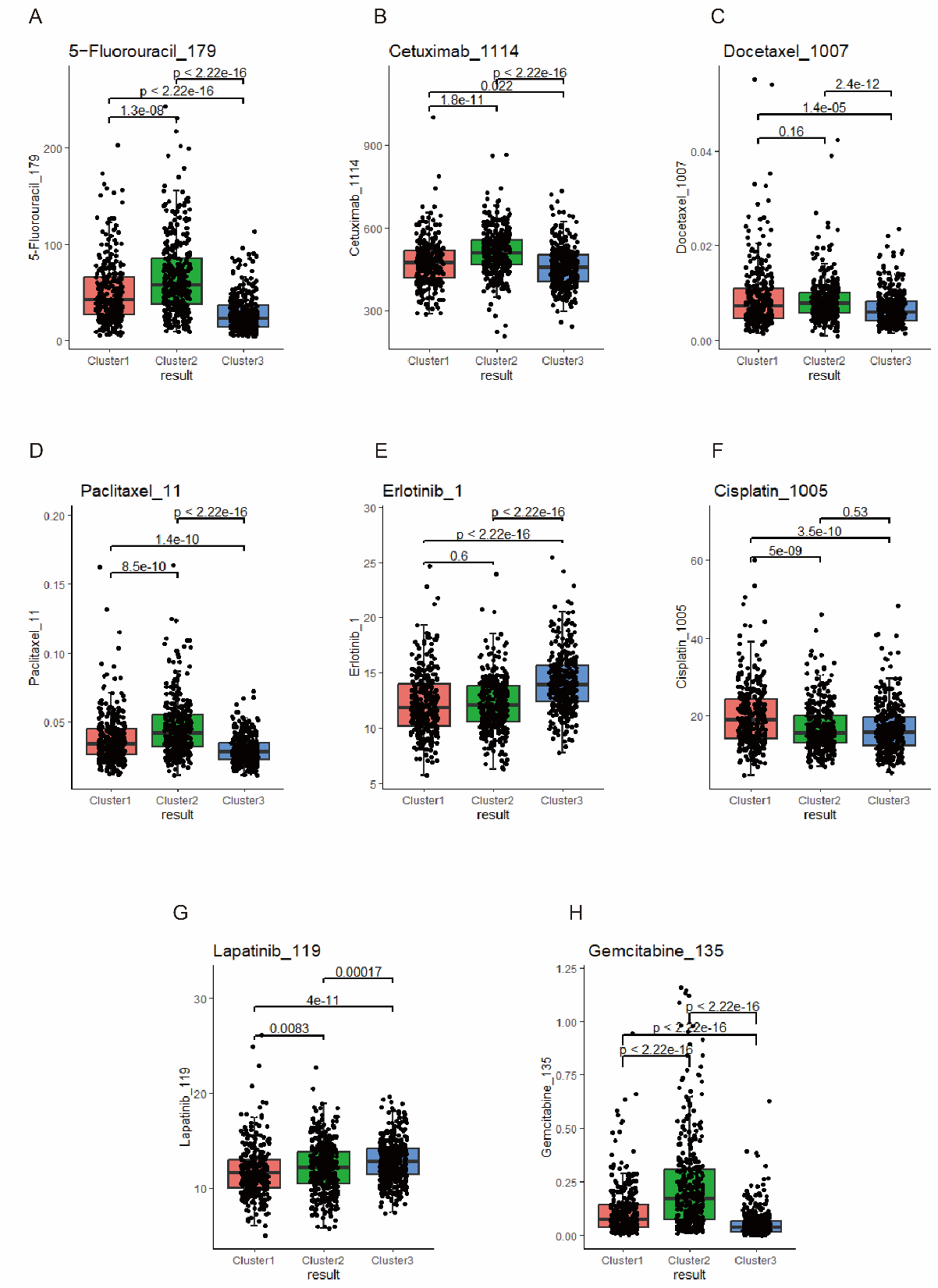


**Supplementary Fig. S6. Predicted chemotherapeutic and targeted drug sensitivity across m⁶A phenotypes.**(A–H) Boxplots comparing the estimated half-maximal inhibitory concentration (IC50) values of eight commonly used chemotherapeutic or targeted agents among the three m⁶A clusters, as predicted by the pRRophetic algorithm based on GDSC data. Drugs include 5-fluorouracil (A), cetuximab (B), docetaxel (C), paclitaxel (D), erlotinib (E), cisplatin (F), lapatinib (G) and gemcitabine (H). Lower predicted IC50 values indicate higher drug sensitivity. P values for pairwise comparisons between clusters are shown above the brackets (Wilcoxon rank-sum test).


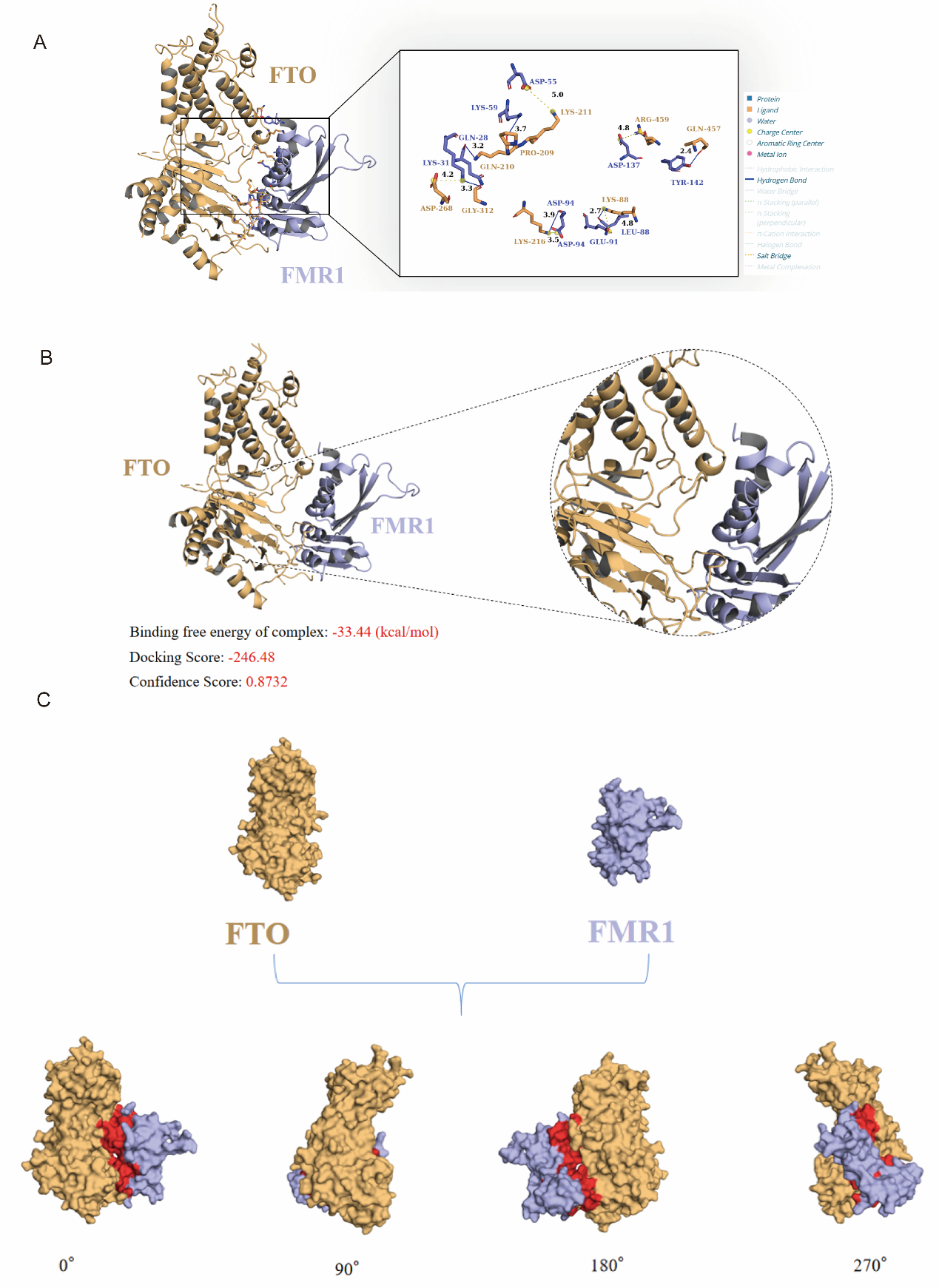


**Supplementary Fig. S7. Molecular docking and structural modeling support the high-affinity interaction of the FMR1–FTO complex.** (A) Ribbon model of the predicted FMR1–FTO complex. The enlarged inset provides a detailed view of the interaction network at the binding interface, highlighting specific amino acid residues and their non-covalent interactions, including hydrogen bonds and salt bridges. (B) Structural interface and quantitative docking metrics of the complex. The calculated binding free energy (-33.44 kcal/mol), docking score (-246.48), and confidence score (0.8732) indicate a thermodynamically favorable and stable binding conformation. (C) Surface representation of FTO, FMR1, and their assembled complex. The complex is displayed at four rotational perspectives (0°, 90°, 180°, and 270°) to visualize the spatial complementarity, with the predicted binding interface highlighted in red.


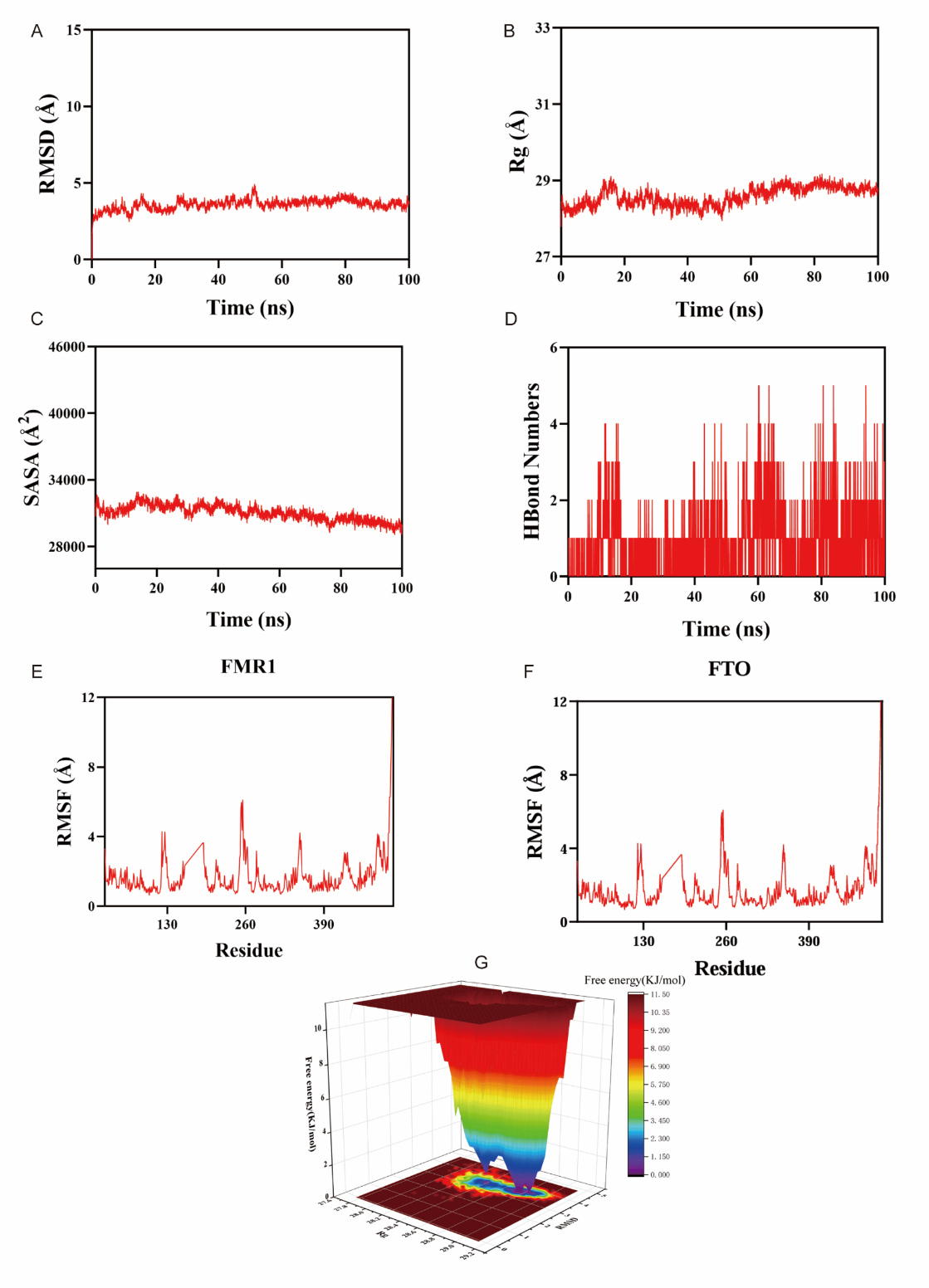


**Supplementary Fig. S8. Molecular dynamics simulations support the structural stability of the FMR1–FTO complex.**(A) Time evolution of the backbone root-mean-square deviation (RMSD) of the FMR1–FTO complex over a 100-ns MD trajectory, showing rapid equilibration and subsequent fluctuations within a narrow range.(B) Time-dependent changes in the radius of gyration (Rg), indicating overall compactness of the complex during the simulation.(C) Solvent-accessible surface area (SASA) of the complex as a function of time, reflecting stable solvent exposure of the protein surface.(D) Number of intermolecular hydrogen bonds between FMR1 and FTO throughout the simulation, demonstrating persistent non-covalent interactions at the binding interface.(E) Residue-wise root-mean-square fluctuation (RMSF) profile of FMR1, highlighting relatively flexible loop regions and a structurally stable core.
(F) Residue-wise RMSF profile of FTO, showing a similar pattern of local flexibility and global rigidity.(G) Three-dimensional free energy landscape of the FMR1–FTO complex derived from the MD trajectory, revealing a dominant low-energy basin consistent with a thermodynamically stable binding conformation.
